# Relaxed sequence constraints favor mutational freedom in idiosyncratic metazoan mitochondrial tRNAs

**DOI:** 10.1038/s41467-020-14725-y

**Published:** 2020-02-20

**Authors:** Bernhard Kuhle, Joseph Chihade, Paul Schimmel

**Affiliations:** 1 The Skaggs Institute for Chemical Biology, Scripps Research, La Jolla, CA 92037 USA; 2 Department of Molecular Medicine, The Scripps Research Institute, La Jolla, CA 92037 USA; 3 Department of Chemistry, The Scripps Research Institute, La Jolla, CA 92037 USA; 4 Department of Chemistry, Carleton College, 1 North College St., Northfield, MN 55057 USA; 5 Department of Molecular Medicine, The Scripps Florida Research Institute, Jupiter, FL 33458 USA

**Keywords:** Biophysical chemistry, RNA, Coevolution, tRNAs, X-ray crystallography

## Abstract

Metazoan complexity and life-style depend on the bioenergetic potential of mitochondria. However, higher aerobic activity and genetic drift impose strong mutation pressure and risk of irreversible fitness decline in mitochondrial (mt)DNA-encoded genes. Bilaterian mitochondria-encoded tRNA genes, key players in mitochondrial activity, have accumulated mutations at significantly higher rates than their cytoplasmic counterparts, resulting in foreshortened and fragile structures. Here we show that fragility of mt tRNAs coincided with the evolution of bilaterian animals. We demonstrate that bilaterians compensated for this reduced structural complexity in mt tRNAs by sequence-independent induced-fit adaption to the cognate mitochondrial aminoacyl-tRNA synthetase (aaRS). Structural readout by nuclear-encoded aaRS partners relaxed the sequence constraints on mt tRNAs and facilitated accommodation of functionally disruptive mutational insults by cis-acting epistatic compensations. Our results thus suggest that mutational freedom in mt tRNA genes is an adaptation to increased mutation pressure that was associated with the evolution of animal complexity.

## Abstract (web summary)

Bilaterian mitochondria-encoded tRNA genes accumulate mutations at higher rates than their cytoplasmic tRNA counterparts, resulting in idiosyncratic structures. Here the authors suggest an evolutionary basis for the observed mutational freedom of mitochondrial (mt) tRNAs and reveal the associated co-adaptive structural and functional changes in mt aminoacyl-tRNA synthetases.

## Introduction

Bioenergetic considerations suggest that mitochondria played a central role in the evolution of eukaryotic complexity^1–3^. Yet, the dependency of eukaryotes on their mitochondria comes at a significant risk. Due to asexual reproduction in small populations, limited recombination, and frequent population bottle-necks, the mitochondrial genome (mtDNA) rapidly accumulates slightly deleterious mutations through random loss of the least-mutated genotypes, a process referred to as Muller’s ratchet^4–6^. In particular bilaterians, with their high aerobic activity and developmental complexity, exhibit exceptionally high rates of mtDNA sequence evolution^7–10^ and are thus at risk of irreversible fitness decline through mtDNA mutational meltdown^4,11–13^. This suggests that bilaterians were under pressure to evolve mechanisms to maintain mitochondrial quality in the face of high substitution rates and the action of Muller’s ratchet^12,14,15^.

Here, we analyzed how the mechanism of genetic code expression in bilaterian mitochondria copes with elevated mutation rates at the molecular level. Throughout cellular life, aaRS-directed matching of tRNA anticodons with cognate amino acids in the aminoacylation reaction serves as a critical check-point for the fidelity of genetic code expression^16–18^. This process critically depends on the aaRS’s ability to strictly discriminate cognate from non-cognate tRNAs. From prokaryotes to the eukaryote cytoplasm, tRNA recognition relies on two major factors: the canonical tRNA fold with its high structural and sequence complexity and thermodynamic stability, and a small fraction of nucleotides, termed identity and anti-identity elements, embedded into the canonical tRNA structural framework^17,19–24^. The importance of this recognition system is highlighted by the strong purifying selection that keeps the canonical tRNA structure and sequence determinants unaltered or only slightly adjusted over billions of years^17,19–24^.

In contrast, during animal evolution, mtDNA-encoded mt tRNAs have undergone an unprecedented metamorphosis. Weak base-pairs and mismatches in stem regions, reduced loop sizes and deletions of entire domains gave rise to “bizarre” mt tRNAs with dramatically reduced thermal stability and low sequence complexity^25–27^. Moreover, previous studies demonstrated a widespread loss of canonical tRNA identity elements for aminoacylation, often retaining only the conserved anticodon^28–34^. Against this background it remains unclear how bilaterian mitochondria achieve the necessary high fidelity of genetic code expression. The importance of mtDNA gene expression for ATP synthesis^14,35^, and the key role of mt tRNA in mitochondrial translation suggest that bilaterians were under pressure to evolve mechanisms that slow down or halt fitness decline due to mutational meltdown^12,14,36,37^. Exemplified by the unique loss of G3:U70 as identity element in the majority of bilaterian mt AlaRS/tRNA^Ala^ recognition systems, we suggest that coadaptation of nuclear-encoded synthetases to their mt tRNA partners altered the recognition rules to allow increased mutational freedom in mt tRNAs to mitigate mutational meltdown.

## Results

### Appearance of unstable mt tRNAs

**Fig. 1:**
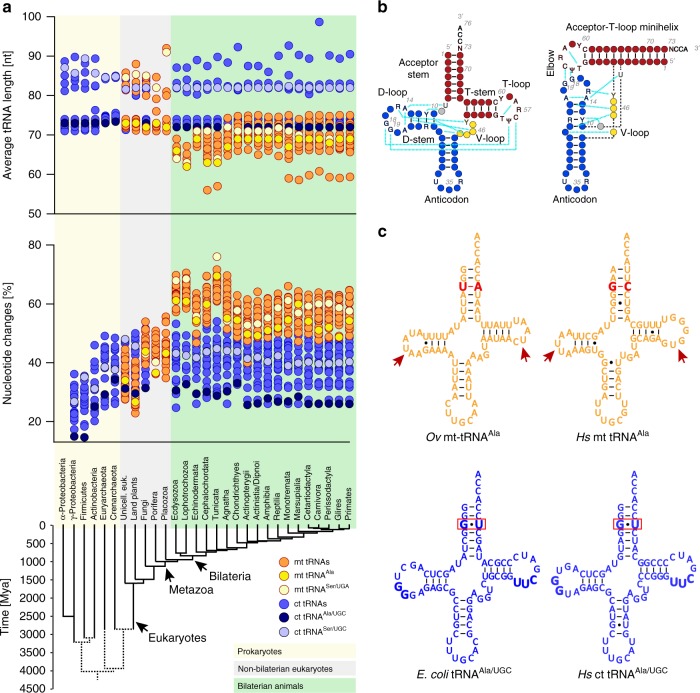
Increased sequence divergence, degeneration of structural determinants and loss of sequence identity elements in bilaterian mt tRNAs. **a** Average length (top) and sequence changes (middle) of mitochondrial (orange) and prokaryotic/cytoplasmic (ct) tRNAs (blue). The average length of ct tRNAs ranges from 72–74 nt to ≥80 nt for tRNAs with long variable arm. While non-bilaterian mt tRNAs retain canonical length distributions, average sequence length of bilaterian mt tRNAs drops significantly (Supplementary Fig. 1b) relative to cytoplasmic counterparts, with ~65 nt (mean) in protostomes (*p* < 0.001) and low branching deuterostomes, and ~69 nt in vertebrates (*p*<0.001). In terms of sequence divergence in tRNAs (middle), non-bilaterian mt tRNAs exhibit moderate but significant increases to ~44% sequence changes compared with ~40% (*p* < 0.001) in ct tRNAs, likely linked to increased A/U content (Supplementary Fig. 1a). Bilaterian mt tRNAs accumulate substitutions further to ~64% and ~58% in protostomes and deuterostomes, respectively, compared with ~40% in ct tRNAs (*p* < 0.001). Again, protostomes and low branching deuterostomes show stronger divergence than vertebrates, possibly also linked to stronger increases in A/U content. Mt tRNA^Ala^ and mt tRNA^Ser/UGA^ are highlighted in dark and light yellow, respectively; ct tRNA^Ala^ and ct tRNA^Ser/UGA^ are highlighted in dark and light blue, respectively. Each data point corresponds to one tRNA identity (Ala, Arg, etc.) within phylogenetic groups indicated below the *x*-axis. Sequence divergence was determined relative to α-proteobacterial tRNAs. The bottom panel shows the phylogenetic relationship and divergence times between the organismal groups for which tRNAs were compared. *P*-values were determined by Wilcoxon rank sum test (Supplementary Fig. 1b). Source data are provided as a Source Data file. **b** Scheme of cloverleaf and L-shaped fold of canonical tRNA with conserved structure- and sequence elements. Canonical tertiary interactions are indicated by cyan dashed lines. **c** Examples of mt tRNA^Ala^ (orange) from *Octopus vulgaris* (*Ov*) and humans (*Hs*) and of canonical tRNA^Ala^ (blue) from *E. coli* and human cytoplasm. Red boxes indicate the G3:U70 identity element. In *O. vulgaris* and human mt tRNA^Ala^, G3:U70 is replaced by U:A and G:C pairs, respectively (bold red) (see also Supplementary Figs. 1–3).

We compared sequences of mitochondrial, eukaryote cytoplasmic, and prokaryotic tRNAs with respect to sequence divergence, length, and nucleotide composition. The analysis showed that mt tRNAs from both major bilaterian phyla, protostomes and deuterostomes, exhibit significantly increased sequence divergence and length variation compared with their cytoplasmic and non-bilaterian mitochondrial counterparts (Fig. 1a and Supplementary Fig. 1). Remarkably, while eukaryote cytoplasmic and non-bilaterian mt tRNAs have 50–70% conserved sites, bilaterian mt tRNAs have only 30–55% that remain constant. Protostome mt tRNAs hereby show a stronger and yet more uniform divergence between 57 and 70%, whereas deuterostome mt tRNAs differ over a wider range from about 45 to 70%. With this insight, we considered the possibility that the idiosyncratic properties of mt aaRS/tRNA systems that are common to bilaterian animals, might be a functional adaptation to specific requirements in bilaterians, in particular the need to deal with high sequence mutation rates in mitochondrially encoded tRNA genes.

### Loss of a major tRNA identity element in bilaterian lineages

We turned to the mt AlaRS/tRNA^Ala^ system to investigate a specific example of how degeneration of mt tRNAs and epistatic changes may be linked to the unique demands of bilaterian mitochondria. From prokaryotes to the human cytoplasm a single acceptor-stem G3:U70 wobble pair marks a tRNA for aminoacylation with alanine^22,24,38,39^. This association is so robust that transplantation of the G3:U70 pair into other, non-cognate tRNAs removes the aminoacylation barrier and confers charging by AlaRS^21,22,24^. Hence, in wide screens, *cis*-acting epistatic mutations that compensated for a missing G3:U70 base pair were difficult to find and, if found, had minimal compensatory effects^23,40^. Comparative analysis of tRNA^Ala^ sequences shows that the G3:U70 identity element and canonical structural determinants are conserved throughout the three domains of life, including non-bilaterian mitochondria. In stark contrast, mt tRNA^Ala^ variants in protostomes and deuterostomes deviate dramatically from their canonical counterparts (Fig. 1 and Supplementary Figs. 2 and 3). Pairwise sequence comparisons show increased accumulation of substitutions and deletions in bilaterian mt tRNA^Ala^ compared with ct tRNA^Ala^ (Fig. 1 and Supplementary Fig. 2). Among mammalian mt tRNA^Ala^ sequences only nine positions are invariant (excluding anticodon and CCA-arm), all of which are presumably involved in core tertiary interactions (Supplementary Fig. 2). At the same time, D- and T-loops are foreshortened (from 7 to 8 and 7 nt, respectively, in canonical tRNA) to 5–7 (average 5.1) and 5–9 (6.9) nucleotides, respectively (Fig. 1c and Supplementary Fig. 2). Moreover, the substitution of more A:U Watson-Crick and G:U wobble pairs (Supplementary Fig. 1a), deletions in loop regions, and loss of canonical structure elements caused a markedly reduced thermal stability (Supplementary Fig. 2c).

Most strikingly, the otherwise invariant G3:U70 identity element is lost from mt tRNA^Ala^ sequences in 20 out of 24 major bilaterian animal phyla, including chordates, while conserved only in nematodes, nematomorphs, hemichordates, and xenacoelomorphs, and shifted to G2:U71 in the arthropod subphylum of insects (Fig. 1c and Supplementary Fig. 3a)^41,42^. Notably, lineages that retain G3:U70 occur in both protostomes and deuterostomes, suggesting that the G3:U70 identity element may have been lost repeatedly in both lineages by convergent evolution (Supplementary Fig. 3a). Bilaterian evolution thus appears to be associated with strong selective pressure to replace G3:U70-dependent recognition alongside the structural degeneration of mt tRNA^Ala^.

### Low sequence complexity is associated with mutational freedom

Focusing on the human system, we asked what alternative mechanisms bilaterian mt AlaRS/tRNA^Ala^ pairs may use to compensate for the loss of G3:U70, and how this may be related to high mutation pressure and elevated substitution rates in mitochondrially encoded tRNA genes.

**Fig. 2:**
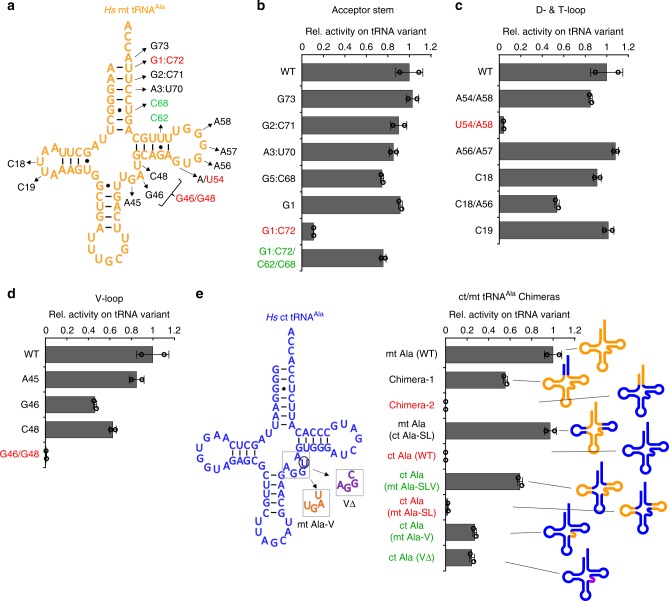
Sequence-independent recognition of *Hs* mt tRNA^Ala^ by mt AlaRS. **a** *Hs* mt tRNA^Ala^ mutants used in charging experiments with mt AlaRS. **b**–**d** Relative charging activities (*k*_cat_/*K*_m_) of mt AlaRS on mt tRNA^Ala^ variants (activity with wild-type mt tRNA^Ala^ is set to 1). **e** Sequence of *Hs* ct tRNA^Ala^ (left) and relative activities of chimeric tRNA^Ala^ variants. Domains are colored orange for mt tRNA^Ala^ and blue for ct tRNA^Ala^. Error bars represent SEM of two independent experiments for each wild-type and mutant. Source data are provided as a Source Data file (see also Table 1).

Consistent with its absence from primate mt tRNA^Ala^ sequences (Fig. 1c and Supplementary Fig. 2d), the human mt AlaRS/tRNA^Ala^ system was recently shown to be insensitive to a G3:U70 identity element^34^. To identify sequence elements within mt tRNA^Ala^ that are used instead, we introduced base-pair substitutions in the acceptor stem, and single or double mutations throughout the D-, T-, and V-loop regions of the tRNA (Fig. 2a–d). Remarkably, we found that the aminoacylation activity of mt AlaRS is virtually insensitive to changes of nucleotides over the entire sequence of mt tRNA^Ala^ (Fig. 2a–d, Table 1).

**Table 1:** Kinetics of aminoacylation of tRNA^Ala^ variants by *Hs* mt AlaRS (related to Fig. 2).

	*k*_cat_	*K*_m_	*k*_cat_/*K*_m_	Rel. *k*_cat_/*K*_m_	Fold change	
	[10^−3^ s^−1^]	[µM]	[10^−3^ s^−1^ µM^−1^]		^a^	^b^
mt Ala(WT)	9.9 ± 0.6	0.46 ± 0.14	21.39 ± 6.50	1.00		
G73	10.6 ± 0.5	0.48 ± 0.1	22.09 ± 4.79	1.03	1	
G2:C71	10.6 ± 0.9	0.55 ± 0.19	19.35 ± 7.03	0.90	1	
A3:U70	10.6 ± 0.4	0.58 ± 0.09	18.31 ± 2.82	0.86	1	
G5:C68	10.3 ± 0.5	0.64 ± 0.13	16.05 ± 3.25	0.75	1	
G1	11.0 ± 0.6	0.56 ± 0.12	19.69 ± 4.31	0.92	1	
G1:C72	4.9 ± 0.2	2.06 ± 0.19	2.38 ± 0.24	0.11	−9	
G1:C72/C62/C68	9.1 ± 0.3	0.56 ± 0.08	16.28 ± 2.26	0.76	1	
A54/A58	9.9 ± 0.5	0.55 ± 0.12	17.90 ± 4.00	0.84	1	
U54/A58	3.1 ± 0.1	3.49 ± 0.28	0.89 ± 0.08	0.04	−24	
A56/A57	9.4 ± 0.6	0.41 ± 0.14	22.80 ± 7.59	1.07	1	
C18	9.3 ± 0.6	0.48 ± 0.15	19.20 ± 6.18	0.90	1	
C18/A56	9.4 ± 0.2	0.83 ± 0.06	11.31 ± 0.78	0.53	−2	
C19	9.6 ± 0.7	0.45 ± 0.15	21.36 ± 7.03	1.00	1	
A45	11.1 ± 0.5	0.61 ± 0.12	18.24 ± 3.60	0.85	1	
G46	11.6 ± 0.2	1.19 ± 0.07	9.77 ± 0.59	0.46	−2	
C48	10.8 ± 0.2	0.82 ± 0.05	13.24 ± 0.82	0.62	−2	
G46/G48	0.8 ± 0.1	4.44 ± 1.01	0.18 ± 0.04	0.01	−120	
ct Ala(WT)	0.1 ± 0.02	5.48 ± 1.68	0.03 ± 0.01	0.001	−790	
ct/mt Chimera-1	6.5 ± 0.2	0.53 ± 0.07	12.11 ± 1.69	0.57	−2	+ 448
ct/mt Chimera-2	0.1 ± 0.02	5.41 ± 1.51	0.03 ± 0.01	0.001	−816	1
mt Ala(ct Ala-SL^c^)	10.4 ± 0.4	0.49 ± 0.08	21.18 ± 3.74	0.99	1	+ 782
ct Ala(mt Ala-SLV^c^)	7.1 ± 0.1	0.47 ± 0.03	15.07 ± 0.92	0.70	1	+ 557
ct Ala(mt Ala-SL^c^)	0.3 ± 0.01	0.57 ± 0.13	0.48 ± 0.11	0.02	−45	+ 18
ct Ala(mt Ala-V^c^)	5.2 ± 0.1	0.88 ± 0.08	5.92 ± 0.53	0.28	−4	+ 219
ct Ala(VΔ^c^)	3.9 ± 0.2	0.73 ± 0.12	5.33 ± 0.90	0.25	−4	+ 197

^a^Loss of activity relative to wild-type mt tRNA^Ala^.

^b^Gain of activity relative to wild-type ct tRNA^Ala^.

^c^S: Stems; L: (D- & T) loops; V: V-loop. Means and standard errors were calculated from two independent experiments. Source data are provided as a Source Data file.

In line with previous results^34^, most substitutions of base-pairs within the acceptor stem had only small effects (≤2-fold decrease in *k*_cat_/*K*_m_). Similarly, aminoacylation was not significantly affected by mutations in the D- or T-loop regions, consistent with the low sequence conservation even among primate mt tRNA^Ala^ variants. Only three (G1:C72, U54/A58, and G46/G48) of the tested mutations (out of 46 changed positions) (Supplementary Fig. 4a) were adverse, with reductions in *k*_cat_/*K*_m_ larger than one order of magnitude. Interestingly, substitutions of A46 and U48 individually had no effect, suggesting that the G46/G48 double mutant interfered with correct folding of the tRNA core. Strikingly, G1:C72 and the U54/A58 double mutant—which introduces a canonical tertiary interaction in the T-loop—could both be recovered by non-reverting mutations (Fig. 2c). Collectively, these results suggest that, consistent with the loss of G3:U70 and low sequence conservation in mt tRNA^Ala^, human mt AlaRS does not use sequence-specific elements in either the acceptor stem or in the loop regions to recognize its cognate mt tRNA substrate.

**Fig. 3:**
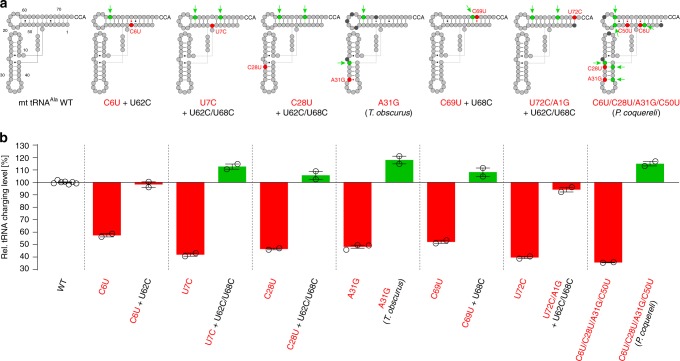
Compensatory epistasis in *Hs* mt tRNA^Ala^. **a** *Hs* mt tRNA^Ala^ is presented in schematic L-shape. Shown is a subset of constructs used in this study that showed compensatory epistasis between sites. Red indicates initial mutations that reduce charging. The positions for compensatory mutations that restore charging are shown in green and are indicated by arrows. “*T. obscurus*” and “*P. coquereli”* denote the mt tRNA^Ala^’s from two primate species that contain known human disease-causing mutations in an overall altered sequence context. Black circles indicate positions with sequence alterations relative to wild-type (WT) *Hs* mt tRNA^Ala^ with unknown effects on stability. **b** Relative charging levels for mt tRNA^Ala^ constructs containing the destabilizing mutation alone or in combination with compensatory mutations. The charging level of wild-type *Hs* mt tRNA^Ala^ is set to 100%. Error bars represent the SEM of two to six independent experiments. Source data are provided as a Source Data file (see also Supplementary Figs. 4–6 and Table 2 for data on the complete set of compensatory mutations used in this study).

To further explore the functional consequences of this mutational freedom in human mt tRNA^Ala^, we investigated the potential for more intricate internal epistatic interactions. Six single-site mutations were separately introduced into stem regions. Four of these are known disease-causing mutations (C6U, C28U, A31G, and C69U), two occupy evolutionarily conserved positions at opposite ends of the acceptor stem (U7C and U72C). These six mutations markedly decreased the overall yields (plateau levels) of charged tRNA, ostensibly because the mutations caused misfolding by further destabilizing the already fragile tRNA (Fig. 3, Table 2, and Supplementary Figs. 4 and 5). All six mutations could be compensated to restore original charging levels by various distant second-site mutations that reintroduced stability into stem regions (Supplementary Figs. 4 and 5). Moreover, *Hs* mt AlaRS efficiently charged mt tRNA^Ala^’s from two other primate species, *Propithecus coquereli* and *Trachypithecus obscurus* (Supplementary Fig. 6), despite the presence of several disease-associated mutations, which individually cause loss of charging in human mt tRNA^Ala^ (Fig. 3, Table 2). The detrimental effects of the disease-causing mutations were thus efficiently compensated by an altered sequence context in these tRNAs (Supplementary Fig. 6). This suggests that, in the sequence space for mt tRNA^Ala^, *Hs* mt AlaRS allows an extended network of functionally neutral ‘neighbors’ which can be accessed by multiple alternative mutational pathways, by either a few large or several smaller steps. Despite apparently strong sensitivity to destabilizing single-site mutations, their effects are conditional (epistatic) on the sequence context in distant parts of the tRNA (Fig. 3, Supplementary Fig. 4). According to this interpretation, the risk of misfolding upon mutation, not the use as identity element, is the most important determinant for the conservation of individual sites in mt tRNA^Ala^ outside of anticodon loop and CCA-arm.

**Table 2:** Charging levels of mt tRNA^Ala^ constructs (related to Fig. 3).

mt tRNA^Ala^ variant	tRNA charged [µM]	tRNA charged relative to WT [%]
*H. sapiens* WT	2.71 ± 0.03	100 ± 1.2
U62C	2.63 ± 0.03	97 ± 1.3
U68C	2.32 ± 0.08	86 ± 3.3
U62C/U68C	2.74 ± 0.05	101 ± 1.9
C6U	1.55 ± 0.05	57 ± 3.0
C6U + U62C	2.66 ± 0.08	98 ± 3.2
C6U + U68C	2.82 ± 0.07	104 ± 2.5
U7C	1.13 ± 0.05	42 ± 4.1
U7C + A66G	2.94 ± 0.07	109 ± 2.5
U7C + U62C	2.03 ± 0.08	75 ± 4.0
U7C + U62C/U68C	3.05 ± 0.08	113 ± 2.6
C28U	1.26 ± 0.02	46 ± 1.7
C28U + G42A	3.01 ± 0.11	111 ± 3.5
C28U + U62C/U68C	2.86 ± 0.12	106 ± 4.3
A31G	1.31 ± 0.05	48 ± 4.2
U39C	1.08 ± 0.02	40 ± 2.2
A31G + U39C	0.93 ± 0.03	34 ± 3.2
A31G + U62C/U68C	1.62 ± 0.07	60 ± 4.3
*T. obscurus*	3.19 ± 0.12	118 ± 3.7
C69U	1.41 ± 0.05	52 ± 3.5
C69U + U62C	2.53 ± 0.11	94 ± 4.3
C69U + U68C	2.93 ± 0.13	108 ± 4.4
C69U + U62C/U68C	2.83 ± 0.06	105 ± 2.1
A1G/U72C	1.07 ± 0.03	40 ± 3.2
A1G/U72C/U62C/U68C	2.55 ± 0.07	94 ± 2.8
C6U/C28U/A31G/C50U	0.97 ± 0.01	36 ± 0.9
*P. coquereli*	3.11 ± 0.07	115 ± 2.3

Charging level of wild-type (WT) *Hs* mt tRNA^Ala^ is set to 100%. The data represent the mean of two to six independent experiments ± standard error.

*T. obscurus*: Corresponds to *Hs* mt tRNA^Ala^ with mutations A31G + G26A/G27A/G54A/G57A/G59A/U62C/C65U.

*P. coquereli*: Corresponds to Hs mt tRNA^Ala^ with mutations C6U/C28U/A31G/C50U + A1G/G5A/U19A/G26A/G27A/U39C/G42A/A53G/G58A/U61C.

Source data are provided as a Source Data file.

### Role of the tRNA elbow

The sequence plasticity of the mt AlaRS/tRNA^Ala^ interaction was further supported in experiments using chimeric tRNA variants, with structural domains swapped between mt tRNA^Ala^ and its cytoplasmic counterpart (Fig. 2e). While mt AlaRS was inactive on ct tRNA^Ala^, the transplantation of the mt-type elbow region (D- and T-stem-loop and V-loop) was sufficient to confer charging on ct tRNA^Ala^. More remarkably, the sole transplantation of the foreshortened mt V-loop (5′-UGAU-3′) or insertion of an unrelated short V-loop (5′-AGGC-3′) into ct tRNA^Ala^ (V-loop 5′-AGGUA-3′) removed the charging barrier. This suggested that the tRNA’s elbow region plays an important role in recognition and that structural context, rather than a specific sequence, is critical for recognition by cognate mt AlaRS.

### Co-adaptive changes in mt AlaRS

**Fig. 4:**
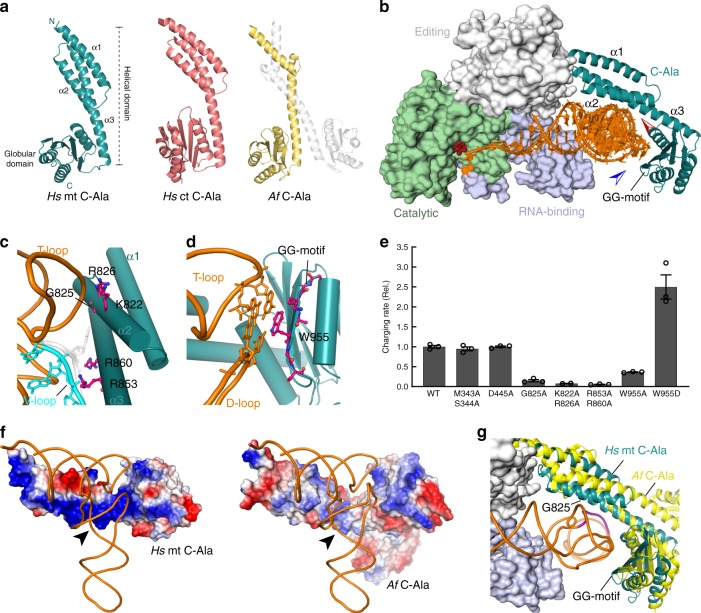
mt tRNA^Ala^ recognition by mt C-Ala. **a** Structures of *Hs* mt C-Ala (left), *Hs* ct C-Ala (middle), and *A. fulgidus* C-Ala (right). **b** Structure model of mt tRNA^Ala^ (orange) bound to *Hs* mt AlaRS. The crystal structure of mt C-Ala is shown in cyan. Red and blue arrows indicate views in (**c**) and (**d**), respectively. **c**, **d** Details of the possible interaction interfaces between mt C-Ala and tRNA as indicated by the model. **c** Interaction of T-stem-loop (orange) and V-loop (cyan) with the N-terminal subdomain. **d** Interaction of T-loop and D-loop (orange) with the globular subdomain. In (**c**), the additional V-loop nucleotide that is absent in mt tRNA^Ala^ but present in ct tRNA^Ala^ (as U47), is shown in transparent gray. **e** Impact of mutations in *Hs* mt AlaRS on mt tRNA^Ala^ charging. Error bars represent SEM of triplicate experiments. Source data are provided as a Source Data file. **f** Comparison of surface charge distributions in *Hs* mt C-Ala (left) and *Af* C-Ala (right). Blue indicates positive, red is negative surface charge. Arrows indicate the position of the V-loop. **g** Superposition of *Hs* mt C-Ala (cyan) and *Af* C-Ala (yellow) based on the C-terminal globular domain (see also Supplementary Figs. 7 and 9; see Supplementary Table 1 for data collection and refinement statistics).

In prokaryotic systems, AlaRS contacts the outside of the V-loop-containing elbow region of the tRNA by using an appended C-terminal (C-Ala) domain^43^. (In *Hs* ct AlaRS, this contact is not required for ct tRNA^Ala^ charging^44^, possibly due to structural incompatibility (Supplementary Fig. 7d–f).) To understand the potential co-adaptive changes promoting elbow-dependent recognition by human mt AlaRS, we solved crystal structures of *Hs* mt C-Ala (Fig. 4a and Supplementary Figs. 7a and 8) and constructed a model of the *Hs* mt AlaRS/tRNA^Ala^ complex based on the *A. fulgidus* AlaRS/tRNA^Ala^ co-crystal structure^38^ (Fig. 4b). The model suggested two major contact sites between mt C-Ala and mt tRNA^Ala^ (Fig. 4c, d). One is in the C-terminal α/β-globular subdomain (aa 873 to C-terminus) containing the glycine-rich “GG” motif (Supplementary Fig. 7b). The second is formed by helices α2 and α3 of the N-terminal subdomain (aa 783–872). Both helices are lined with highly conserved basic side chains that form an extended patch of positive surface charges (Fig. 4f). Due to the 3-helix architecture, helices α2 and α3 can move significantly closer to the tRNA than in the prokaryotic complex, allowing direct contacts with the T-stem and V-loop (Fig. 4g). In particular, the mt AlaRS-specific Gly825 appears to be important for tight association between C-Ala and tRNA and may explain the sensitivity to V-loop size (see above). Consistently, charging was severely affected by mutations that substituted conserved residues in the corner-binding region (Fig. 4e).

It is noteworthy that the recognition dyad of Asn and Asp (Asn317 and Asp416 in *Hs* ct AlaRS) that recognizes the signature G3:U70 pair in canonical AlaRS systems was lost during bilaterian mt AlaRS evolution and replaced by other amino acid side chains (Fig. 4e and Supplementary Figs. 7c and 9), such as Met343 (for Asn317) and Gly444 (for Asp416) in human mt AlaRS^34^. Thus, adaptive evolution of the *Hs* mt AlaRS/tRNA^Ala^ recognition system was accompanied by a radical reorganization of its recognition system, moving the center for tRNA recognition from sequence-specific (G3:U70) readout in the acceptor stem to C-Ala-mediated sequence-unspecific readout of the tRNA’s elbow structure (Supplementary Fig. 7g).

### Discrimination against non-cognate mt tRNAs

**Fig. 5:**
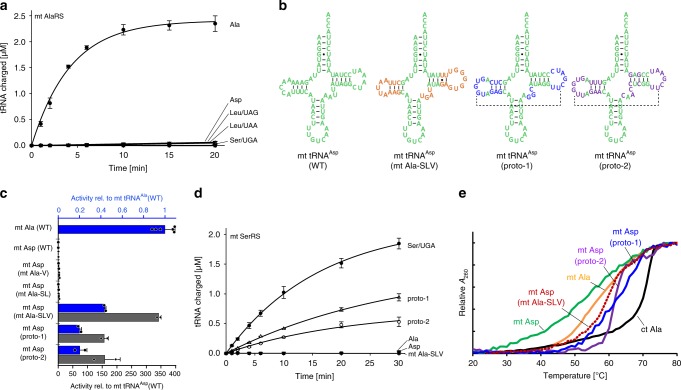
Structural idiosyncrasies and instability in the tRNA elbow region act as anti-identity elements between mtaaRS/tRNA systems. **a** *Hs* mt AlaRS charges cognate mt tRNA^Ala^ but not mt tRNAs specific for Asp, Leu, or Ser. Error bars represent the SEM from triplicate experiments. **b** Cloverleaf presentations of *Hs* mt tRNA^Asp^ (left) and variants containing D- and T-stem-loop and V-loop elements from mt tRNA^Ala^ (orange) or canonical ct tRNAs (blue/purple). Dashed lines indicate the potential for canonical D-loop-T-loop tertiary interactions. **c** Charging activity of *Hs* mt AlaRS on mt tRNA^Asp^ variants relative to activities on wild-type mt tRNA^Ala^ (blue; top *y*-axis) and wild-type mt tRNA^Asp^ (gray; bottom *y*-axis). See also Table 3. Error bars represent SEM of two to six independent experiments. Source data are provided as a Source Data file. **d** *Hs* mt SerRS charges cognate mt tRNA^Ser/UGA^ and stabilized mt tRNA^Asp^ variants but not wild-type mt tRNAs specific for Ala or Asp. Error bars represent the SEM from triplicate experiments. **e** Thermal melts for wild-type and stabilized mt tRNA variants and *Hs* ct tRNA^Ala^ (see also Supplementary Fig. 10).

In view of the plasticity of the *Hs* mt tRNA^Ala^ sequence for recognition by *Hs* mt AlaRS, we next investigated how mt AlaRS discriminates against non-cognate mt tRNAs. For this purpose, we selected four *Hs* mt tRNAs (Asp, Leu(UAG), Leu(UAA), Ser(UGA)), three of which have 4 nt V-loops that should be compatible with the C-Ala domain of *Hs* mt AlaRS (Supplementary Fig. 10). None of them were charged by *Hs* mt AlaRS, indicating efficient discrimination (Fig. 5a, Table 3). Notably, all four were equally unstable or less stable than *Hs* mt tRNA^Ala^ (Supplementary Fig. 10a). Prior work showed that the tertiary interactions needed to form the elbow (comprised of elements from the D-, T-, and V-loops) of the L-shaped tRNA is the least stable part of the structure and disengages first when the tRNA is subjected to thermal melting^45^. This suggests that the ‘elbow’ of each tRNA was loose and possibly not immediately adapted into the C-Ala pocket, thereby preventing charging. It is noteworthy that, although post transcriptional modifications can improve mt tRNA stabilities, they do not completely compensate for their structural weakness. Indeed, melting temperatures of native (modified) mt tRNAs are 10–20 degrees lower than for their cytoplasmic counterparts^26,46,47^. In all systems tested here, aminoacylation of the unmodified wild-type mt tRNAs was robust and retained specificities, suggesting that modifications were not in general connected to tRNA identities.

**Table 3:** Kinetics of aminoacylation of tRNA^Asp^ variants by *Hs* mt AlaRS (related to Fig. 5).

	*k*_cat_	*K*_m_	*k*_cat_/*K*_m_	Rel. *k*_cat_/*K*_m_	Rel. *k*_cat_/*K*_m_
	[10^−3^ s^−1^]	[µM]	[10^−3^ s^−1^ µM^−1^]	(tRNA^Asp^)^a^	(tRNA^Ala^)^b^
mt Ala(WT)	9.9 ± 0.6	0.46 ± 0.14	21.39 ± 6.50		1
mt Asp(WT)	0.3 ± 0.1	11.7 ± 5.4	0.03 ± 0.01	1	
mt Asp(mtAla-V^c^)	0.8 ± 0.2	7.7 ± 4.6	0.10 ± 0.06	4	0.005
mt Asp(mtAla-SL^c^)	0.1 ± 0.02	1.8 ± 1.1	0.07 ± 0.04	3	0.003
mt Asp(mtAla-SLV^c^)	6.7 ± 0.2	0.7 ± 0.1	9.39 ± 1.50	355	0.44
mt Asp(proto-1)	3.0 ± 0.1	0.7 ± 0.1	4.18 ± 0.58	158	0.20
mt Asp(proto-2)	3.6 ± 0.2	0.8 ± 0.2	4.28 ± 0.84	162	0.20

^a^Activity relative to wild-type mt tRNA^Asp^.

^b^Activity relative to wild-type mt tRNA^Ala^.

^c^S: Stems; L: (D- & T) Loops; V: V-loop. Means and standard errors were calculated from two to six independent experiments. Source data are provided as a Source Data file.

To investigate whether mt AlaRS indeed acts in a chaperone-like manner to recognize the fragile elbow region of its cognate tRNA, we altered a non-cognate mt tRNA to obtain increased corner stability. We selected mt tRNA^Asp^ which is identical to mt tRNA^Ala^ in the first three acceptor-stem pairs and shares similar sequences and sizes in its loop regions. When we transplanted the V-loop, D- and T-stem-loops from mt tRNA^Ala^ into mt tRNA^Asp^, the thermal stability of the corner region was enhanced, as judged by the thermal melting profile (Fig. 5e). This increase in stability coincided with the appearance of aminoacylation activity (Fig. 5c, Table 3). Evidently, the idiosyncrasies in the elbow region, not in the acceptor stem, determine the tRNA identities that allow AlaRS to recognize mt tRNA^Ala^ and to discriminate against mt tRNA^Asp^. Moreover, we introduced into mt tRNA^Asp^ either of two unrelated elbow regions that were selected from different prototypical ct tRNA formats (in each case the V-loop was reduced to 4 nt). Both constructs, tRNA^Asp^/proto-1 and tRNA^Asp^/proto-1, conferred greater stability and, again, aminoacylation with mt AlaRS was achieved (Fig. 5, Table 3).

### Sequence-independent readout of mt tRNA^Ser^ by human mt SerRS

As for the mt AlaRS/tRNA^Ala^ system, bilaterian mt SerRS/tRNA^Ser^ systems have lost canonical identity elements, namely the long variable arm (Supplementary Fig. 3b). Instead, bovine mt SerRS was shown to use contacts in the elbow region of its tRNA substrates^28,29^. To determine whether this dependency involves, as for mt AlaRS, sequence-unspecific structural recognition of the corner, we tested *Hs* mt SerRS on various mt tRNAs, mt tRNA^Asp^/proto-1 and tRNA^Asp^/proto-2. While non-cognate tRNA^Asp^ and tRNA^Ala^ were not charged, mt tRNA^Ser/UGA^ and both tRNA^Asp^ variants with prototypical elbow regions were readily aminoacylated (Fig. 5d), demonstrating that mt SerRS also uses the unique structural characteristics of the elbow region to discriminate against fragile non-cognate mt tRNAs.

Importantly, previous studies found similar characteristics in other mammalian mt aaRS/tRNA recognition systems^30–33,48^. *Hs* mt LeuRS gained a chaperone-like activity to support on-enzyme folding of partly unfolded mt tRNA^Leu^ in vitro and to protect destabilized mt tRNA^Leu^ in vivo, while at the same time losing sensitivity to nucleotide substitutions in the acceptor stem^30,31,49^. Similarly, mammalian mt TyrRS evolved insensitivity to the normally critical first acceptor-stem pair (1:72) in mt tRNA^Tyr^, a highly conserved tyrosine identity element in canonical recognition systems^32^. Moreover, human mt tRNA^Asp^ lost conserved identity elements in the acceptor stem and tertiary interactions between D- and T-loop. Still, *Hs* mt AspRS efficiently recognizes mt tRNA^Asp^, ostensibly through interactions with the anticodon^33,50^. Hence, mitochondrial synthetases, in stark contrast to canonical recognition systems, compensate for the intrinsic fragility in their mt tRNA substrates, most likely by using increased electropositive surface potentials in their binding interfaces with tRNA^29,51–53^ (Fig. 4f). This contrast is further highlighted by the strict unilateral aminoacylation barrier between the two systems that allows mammalian mt aaRSs to aminoacylate canonical tRNAs, while canonical aaRSs are generally unable to aminoacylate even cognate mt tRNA species^48,54^.

## Discussion

We studied the mitochondrial aminoacylation system to understand how genetic code expression copes with the elevated mutation pressure in bilaterian mitochondria. Our results suggest that bilaterian mt aaRS/tRNA recognition systems are the product of extensive coadaptation between nuclear-encoded synthetases and their mt tRNA substrates, allowing more relaxed functional constraints on the sequences of rapidly evolving mtDNA-encoded tRNA genes.

This co-adaptive process is exemplified by the human mitochondrial alanine system. In previously studied cases from prokaryotes to the human cytoplasm, tRNA^Ala^ is recognized by AlaRS using a single G3:U70 in the acceptor stem, which epitomizes the principles of canonical recognition based on unique sequence identity elements embedded into the universal tRNA structural scaffold. This identity element, along with canonical structural elements, was lost from mt tRNA^Ala^’s in the majority of bilaterian mitochondrial aminoacylation systems. For the human mt AlaRS/tRNA^Ala^ system we show that loss of the G3:U70 identity element was accompanied by a radical reorganization of the recognition mechanism, away from sequence-specific direct readout in the acceptor stem to sequence-unspecific structural readout of the flexible tRNA elbow. The appended C-Ala domain, which plays an auxiliary role in prokaryotic AlaRSs and is dispensable for aminoacylation in the human cytoplasmic AlaRS system^43,44^, plays a central role as a folding aid for the intrinsically flexible mt tRNA elbow. Fine-tuned induced-fit adaptation selects mt tRNA^Ala^ and rejects non-cognate mt tRNAs (Supplementary Fig. 7g). Similarly, we show that mammalian mt SerRS, which uses sequence-unspecific readout to recognize the two highly divergent mt tRNA^Ser^ isodecoders^28^, discriminates against non-cognate tRNAs based on the incompatibility of their flexible, non-canonical elbow structures. Together with previous work^28,30,31,33,51,54^, these results suggest that relaxed sequence constraints on mt tRNAs are a common characteristic among mammalian mt aaRS/tRNA systems, with mt aaRSs compensating for the intrinsic fragility in their cognate mt tRNA^29,51–53^.

The principles operating in mt aaRS/tRNA recognition systems are thus conceptually related and may have their evolutionary origin in the capacity of canonical aaRS/tRNA systems for indirect readout^55^. Yet, like direct readout of sequence elements, indirect readout in canonical aaRS/tRNA systems depends on the canonical tRNA fold and strong sequence conservation^55,56^. By contrast, mt aaRS/tRNA systems achieve unique tolerance toward the high mutational burden in mtDNA by using the mt tRNAs’ low complexity and idiosyncratic structural features as (anti-) identity elements to discriminate cognate from non-cognate tRNAs (Supplementary Fig. 10c, d).

Ct tRNA import and use in mitochondrial translation is widespread in non-bilaterians such as protozoans, plants, and fungi, where it is associated with canonical recognition systems and usually coincides with the loss of tRNA genes from the mtDNA genome^26,57,58^. Although the capacity for ct tRNA^Gln^ import into human mitochondria has been reported^59^, its physiological relevance remains controversial^37,60,61^. (Humans lack a dedicated mt GlnRS, instead relying on an indirect transamidation pathway catalyzed by mt GluRS and the essential mt GatCAB complex^62^. Because ct tRNA^Gln^ is a poor substrate for the mt GatCAB complex^62,63^, it seems unlikely that ct tRNA^Gln^ is used in normal mitochondrial translation.) According to our results, the risk of misaminoacylation by mt aaRSs argues against the systematic use of imported ct tRNAs in human mitochondrial translation. The strong conservation of minimal mt tRNA sets among bilaterian species and the occurrence of disease-causing mutations in human mt tRNAs (including mt tRNA^Gln^), suggests that functional insufficiencies in mt tRNAs are not compensated by the import of redundant ct tRNA functions in vivo. Importantly, this does not exclude the possibility that ct tRNA import into mitochondria plays a role in adaptive mistranslation^64^, or other, non-translational functions.

The evolution of “bizarre” mt tRNAs, lacking canonical structural determinants and sequence identity elements, is not common among eukaryotes. While mt tRNAs from fungi and non-bilaterian metazoans show higher A/U content and increased substitutions compared with ct tRNAs, “bizarre” mt tRNAs are specific to and universal among bilaterian lineages (Fig. 1 and Supplementary Figs. 1–3). A unique pressure to reorganize mitochondrial aminoacylation systems thus appears to be linked to the evolution of increased complexity in animals, which was accompanied by elevated tissue turnover, higher metabolic demands and increased mitochondrial replication errors^12,14^

We suggest that the widespread loss of canonical sequence determinants, down-sizing and structural instability in bilaterian mt tRNAs in combination with the co-adaptive remodeling of their mt aaRS interactions, may be viewed as a molecular adaptation to preserve mitochondrial fitness in the face of elevated mutation pressure and Muller’s ratchet. An important characteristic of the mitochondria-specific recognition mechanism is the reduced functional constraint that acts on mt tRNA sequences compared with canonical systems. A recognition mechanism that uses shape and folding properties rather than atomic determinants to discriminate cognate from non-cognate tRNA substrates and relies on nuclear-encoded mt aaRSs to support tRNA folding increases the functionally neutral sequence space in mt tRNAs to compensate mutational insults by epistatic second-site mutations. The consequences are epistatic fitness ridges along which mt tRNAs can evolve rapidly by neutral or near-neutral mutations^65,66^, without inevitably leading to irreversible functional degeneration or mutational meltdown.

## Methods

### mt AlaRS constructs

The gene encoding *Hs* mt-AlaRS (comprising residues 31-C) was cloned into NdeI/XhoI sites of pET-21b vector (Novagen). The final expression construct lacks the coding region for the N-terminal 30 amino acids, corresponding to the cleaved mitochondrial targeting signal and encodes an additional C-terminal [6x]His-Tag. *E. coli* BL21(DE3) cells containing the *Hs* mt AlaRS plasmid were grown in LB medium at 37 °C to an OD of 0.7. Cultures were then allowed to cool down to room temperature (23 °C) and expression was induced by addition of IPTG at a final concentration of 0.4 mM. Cells were harvested after 16 h at RT. *Hs* mt-AlaRS was purified by Ni-NTA beads (Qiagen), followed by a HiTrap Heparin column (GE Healthcare), and a HiLoad 16/60 Superdex 200 column (GE Healthcare) equilibrated in 20 mM Tris pH 7.5, 200 mM NaCl, 5% Glycerol, and 1 mM DTT. Purified *Hs* mt AlaRS was concentrated and stored at −80 °C. Mutant proteins were constructed by site-directed mutagenesis and purified using the same method. The quality of each protein purification was validated by SDS-PAGE analysis.

The gene coding for *Hs* mt SerRS was cloned into BamHI/XhoI sites of pGEX-6P1 vector. Expression and purification procedures were essentially the same as for mt AlaRS, with the exception that GSH-sepharose beads were used in the initial affinity purification step instead of Ni-NTA.

Constructs of *Hs* mt C-Ala were cloned into BamHI/XhoI sites of pGEX-6P1 vector (Amersham). All three constructs were transformed into *E. coli* BL21(DE3) cells and expressed as N-terminal GST-fusions as described above. Fusion proteins were first purified by GSH-sepharose beads (GE Healthcare), followed by the removal of the GST-tag by Prescission Protease (GE Healthcare) digest. The cleaved protein was further purified by HiTrap Heparin column, and a HiLoad 16/60 Superdex 200 column equilibrated in 10 mM HEPES pH 7.5, 100 mM NaCl, and 1 mM DTT. Purified C-Ala constructs were concentrated to 10–15 mg/mL and stored at −80 °C.

### Crystallization and X-ray data collection

Initial high-throughput crystallization screens were performed by the sitting-drop vapor diffusion method using a Mosquito liquid transfer robot (TTP Labtech). Each drop contained 200 nL of 10–15 mg/mL protein and 200 nL of reservoir solution and was equilibrated against 40 μL of reservoir solution. For mt AlaRS(873-C), single crystals grew after 1 day at 23 °C in 0.1 M Tris/HCl (pH 7.0), 300 mM CaCl_2_, 18% PEG 3350. Single crystals for mt AlaRS(802-C) were obtained in 0.1 M Tris (pH 8.5), 40% PEG 200 after 2 days at 23 °C. Finally, clusters of rod-shaped crystals of mt AlaRS(783-C) were obtained over night at 4 °C in 0.1 M Imidazole (pH 7.0), 2 M NaCl. Crystals of mt AlaRS(873-C) and mt AlaRS(783-C) were cryoprotected with 10–15% (v/v) glycerol added to the reservoir solution and flash-frozen in liquid nitrogen. X-ray diffraction data were collected on beamline 12-2 at the Stanford Synchrotron Radiation Lightsource (SSRL) at 100 K and a wavelength of 0.9740 Å. Diffraction images were processed with the XDS package^67^. X-ray diffraction data statistics are summarized in Supplementary Table 1.

### Structure determination and refinement

Human mt AlaRS(873-C) crystallized in space group P2_1_ with 4 molecules per asymmetric unit (AU). The phase problem was solved by the molecular replacement (MR) method using PHASER^68^. The globular domain of human cytoplasmic C-Ala was used as search model. Model building and refinement were performed in iterative cycles using Coot^69^ and PHENIX^70^. The final mt AlaRS(873-C) model was refined to 1.15 Å resolution with *R*_work_ = 15.85% and *R*_free_ = 17.23% (Supplementary Fig. 8). Crystals of mt AlaRS(802-C) grew in space group C222_1_ with 4 molecules per AU. The phase problem was solved by MR using the structure of mt AlaRS(873-C) as search model. The missing helical region N-terminal to the globular domain was built manually in Coot, followed by reiterated refinement in PHENIX. The final mt AlaRS(802-C) model was refined to 2.23 Å resolution with *R*_work_ = 20.94% and *R*_free_ = 25.7%. mt AlaRS(783-C) crystals grew in space group I4_1_ with two molecules per AU. The phase problem was solved by MR using mt AlaRS(873-C) and the N-terminal portion of mt AlaRS(802-C) for the search. Placement of helix 1 was performed in Coot. The final model of mt AlaRS(873-C) was refined to 4.09 Å resolution with *R*_work_ = 22.32% and *R*_free_ = 28.2%. A summary of refinement statistics is given in Supplementary Table 1.

### Structural modeling

The homology model of full-length *Hs* mt AlaRS bound to mt tRNA^Ala^ was generated based on the crystal structure of the *A. fulgidus* AlaRS/tRNA^Ala^ complex and the crystal structure of human cytoplasmic AlaRS in combination with sequence alignments. The crystal structure of *Hs* mt C-Ala was placed by superposition of its C-terminal globular domain onto the globular domain of C-Ala in the *A. fulgidus* AlaRS/tRNA^Ala^ complex that interacts with the elbow region of tRNA^Ala^. Without further adjustments, this places the N-terminus of the helical domain in direct vicinity to the C-terminus of the editing domain. *Hs* mt AlaRS was modeled as a monomer. As for human cytoplasmic AlaRS^44^, the three-helix architecture in the N-terminal subdomain of C-Ala prevents dimer formation as observed for prokaryotic AlaRSs. This is consistent with the crystal structure analysis of mt C-Ala, as well as with size exclusion chromatography experiments.

### In vitro transcription of tRNAs

The various tRNA genes used in this study were either cloned into pUC-19 vector or purchased as synthetic oligos with the tRNA-coding region preceded by a hammerhead (HH) ribozyme under the control of a T7 RNA polymerase promoter. Mutant genes were generated by site-directed mutagenesis following the QuikChange protocol (Stratagene). All DNA-templates for in vitro transcription were amplified by PCR using forward and reverse primers complimentary to the T7 promoter and the 3′ end of the tRNA gene, respectively. Run-off transcription reactions were performed in 40 mM Tris-HCl pH 8.0, 25 mM NaCl, 25 mM MgCl_2_, 2 µg/mL pyrophosphatase, 1 mM Spermidine, 5 mM DTT, 4 mM of ATP, CTP, GTP, and UTP with 75 µg/mL T7 polymerase and DNA template at 37 °C for 3 h. After transcription, the RNA was incubated for 1 h at 60 °C to enhance the autocatalytic cleavage of the HH transzyme. Reactions were stopped by phenol/chloroform extraction followed by purification of cleaved tRNA by 12% denaturing PAGE. tRNA was eluted from the gel in buffer containing 200 mM NaOAc, 20 mM Tris/HCl, 5 mM EDTA (pH 5.3), and annealed by first heating to 80 °C, followed by gradual cooling to 20 °C at a rate of 2°/min. The tRNA was finally ethanol-precipitated, taken up in RNase-free water and stored in small aliquots at −80 °C.

### Active site titration assay

The concentration of active sites was determined at room temperature (25 °C) in 40-μl reactions containing two different concentrations (5 and 10 μM) of *Hs* mt aaRS (AlaRS or SerRS), 20 mM L-amino acid, 22 nM [γ-^32^P]-ATP, in assay buffer (100 mM Hepes pH 7.5, 20 mM KCl, 10 mM MgCl_2_, 2 mM DTT, and 2 mg/mL pyrophosphatase). Reactions were initiated by adding enzyme to the assay solution in 96-well low-profile PCR plates. At different time points 5 μl reaction mix were quenched into PVDF MultiScreen filter plates (0.45 μm pore size hydrophobic, low-protein-binding membrane; Merck Millipore) containing 20 μl of 7% HClO_4_ and 80 μl of 10% charcoal slurry. Following the last time point, the slurry was mixed by pipetting and centrifuged into a 96-well flexible PET microplate (PerkinElmer) containing 150 μL of Supermix scintillation mixture (PerkinElmer). The plate was counted on a 1450 MicroBeta Micoplate Scintillation and Luminescence Counter (PerkinElmer).

### In vitro aminoacylation

For *Hs* mt AlaRS aminoacylation reactions were carried out in an assay solution containing 50 mM Hepes pH 7.5, 30 mM KCl, 7.5 mM MgCl_2_, 4 mM ATP, 2 mM DTT, 4 μg/mL pyrophosphatase, 20 μM cold l-alanine, and 1.34 μM [^3^H]-alanine (1 mCi/mL). Reactions with *Hs* mt SerRS were performed in an assay solution containing 50 mM Hepes pH 7.5, 60 mM KCl, 10 mM MgCl_2_, 4 mM ATP, 5 mM DTT, 4 μg/mL pyrophosphatase, 1 mM Spermine, 10 μM cold l-serine, and 5 μM [^3^H]-serine (1 mCi/mL). Varying amounts of tRNA were initially mixed with assay solution, and the reaction was initiated by addition of *Hs* mt AlaRS or *Hs* mt SerRS (0.4 or 1 µM). At varying time intervals, 5-μL aliquots were removed and applied to a MultiScreen 96-well filter plate (0.45 μm pore size hydrophobic, low-protein-binding membrane; Merck Millipore), pre-wetted with quench solution (0.5 mg/mL salmon sperm DNA, 0.1 M EDTA, 0.3 M NaOAc (pH 3.0)). After all time points were collected, 100 μL of 20% (w/v) trichloroacetic acid (TCA) was added to precipitate nucleic acids. The plate was then washed four times with 200 μL of 5% TCA containing 100 mM cold alanine, followed once by 200 μL of 95% ethanol. The plate was then dried, followed by deacylation of bound tRNAs by addition of 70 μL of 100 mM NaOH. After 10 min incubation at RT, the NaOH-solution was centrifuged into a 96-well flexible PET microplate (PerkinElmer) with 150 μL of Supermix scintillation mixture (PerkinElmer). After mixing, the radioactivity in each well of the plate was measured in a 1450 MicroBeta Micoplate Scintillation and Luminescence Counter (PerkinElmer).

### Thermal melting assay

To monitor thermal stability of tRNAs, temperature dependent UV melting experiments were carried out using a Cary 100 (Varian) Spectrophotometer equipped with temperature-controlled cell holder. A quartz cell of 1-cm path length was used for all absorbance studies. Temperature dependent absorption spectra were obtained at 260 nm with temperature increasing at a rate of 0.5 °C/min from 20 to 80 °C. The concentration of tRNAs was 1 μM. The buffer solution contained 10 mM sodium cacodylate buffer, 0.1 mM EDTA and 70 mM NaCl at pH 7.5.

### tRNA sequence analysis

Sequences for genes encoding mitochondrial, prokaryotic or eukaryote cytoplasmic tRNAs were retrieved from tRNAdb/mitotRNAdb (http://trna.bioinf.uni-leipzig.de/), the genomic tRNA database (GtRNAdb; http://gtrnadb.ucsc.edu/), and GenBank/NCBI (https://www.ncbi.nlm.nih.gov/genbank/). Sequence alignments of tRNA genes were performed using the ClustalW function of the Molecular Evolutionary Genetics Analysis (MEGA 7.0) software^71^. Misaligned regions were curated manually based on structural characteristics of the tRNAs (i.e., corrected for alignments of stem- and loop regions). The CCA end, which is encoded in subsets of prokaryotic tRNA genes, was removed from the alignments and excluded from subsequent analyses of sequence divergence or sequence length. Sequences of mt tRNA that were too divergent to be aligned with high confidence, usually due to frequent parallel loss of entire structural domains (e.g., mt tRNA sequences from nematodes and individual mt tRNAs from arachnids, mollusk, and tunicate species) were removed from the alignments and excluded form analysis. For the analyses shown in Fig. 1a and Supplementary Fig. 1b, tRNAs of a given specificity (Ala, Arg, Asn, Asp, Cys, Gln, Glu, Gly, His, Ile, Leu, Lys, Met, Phe, Pro, Ser, Thr, Trp, Tyr, Val) were grouped based on phylogeny (including Alphaproteobacteria, Gammaproteobacteria, Bacteroidetes, Firmicutes, Actinobacteria, Euryarchaeota, Crenarchaeota, Land plants, Protozoa (unicellular eukaryotes), Fungi, Porifera, Placozoa, Ecdysozoa (Nematoda (not for mt tRNAs), Crustacea, Insecta, Lophotrochozoa (Annelida, Brachiopoda, Mollusca), Echinodermata, Cephalochordata, Hemichordata, Tunicata, Agnatha, Chondrichthyes, Actinopterygii (Chondrostei, Elopomorpha, Euteleostei), Actinistia, Dipnoi, Amphibia, Reptilia (Aves, Crocodilia, Testudines, Lepidosauria), Monotremata, Marsupialia, Cetartiodactyla (Artiodactyla, Cetacea), Carnivora, Perissodactyla, Glires (Lagomorpha, Rodentia), Primates (Strepsirrhini, Platyrrhini, Catarrhini)). Sequence distance estimates (combining transitions, transversions, and insertions/deletions) between groups were done using the MEGA (MEGA 7.0) software^71^. For Analyses shown in Fig. 1a and Supplementary Fig. 1b, Alphaproteobacteria, as the group most closely related to the likely bacterial ancestors of mitochondria, were chosen as reference group for comparison. Statistical significance of sequence divergences and length differences between mitochondrial and cytoplasmic tRNAs were assessed by Wilcoxon rank sum test (two-tailed). All test statistics were calculated in GraphPad Prism (Prism8).

The relative A/U content of tRNAs shown in Supplementary Fig. 1a was calculated from all annotated tRNA sequences available for the indicated phylogenetic groups.

For Supplementary Fig. 2b, mt tRNA and ct tRNA sequences from Old World monkeys (Catarrhini) were used as reference group for comparison with tRNAs from other metazoan groups. Estimates for divergence times between lineages shown in the bottom panels of Fig. 1a and Supplementary Fig. 1a were obtained from Timetree (http://www.timetree.org/) and references therein.

### Reporting summary

Further information on research design is available in the Nature Research Reporting Summary linked to this article.

## Supplementary information

Supplementary Information

Peer Review

Reporting Summary

## Data Availability

All data generated or analyzed during this study are either included in this published article (and its supplementary information file) or are available from the corresponding author upon request. Structure coordinates were deposited in the PDB with accession codes 6NLQ for mt C-Ala(873–985), 6NLY for mt C-Ala(802–985), and 6NOW for mt C-Ala(783–985). The source data underlying Figs. 1a, 2b–e, 3b, 4e and 5c and Supplementary Figs. 1a, b and 4c are provided as a Source Data file.

## Source data

Source Data
