# Supplementary material for: Relaxed sequence constraints favor mutational freedom in idiosyncratic metazoan mitochondrial tRNAs
Source: Nat Commun. 2020 Feb 20;11:969. doi: 10.1038/s41467-020-14725-y (PMC7033119; doi:10.1038/s41467-020-14725-y)
Supplement: Supplementary file 1 — Supplementary Information [file 41467_2020_14725_MOESM1_ESM.pdf]

Supplementary Information

Kuhle et al.

Relaxed sequence constraints favor mutational freedom in idiosyncratic metazoan mitochondrial tRNAs

Supplementary Figures and Tables

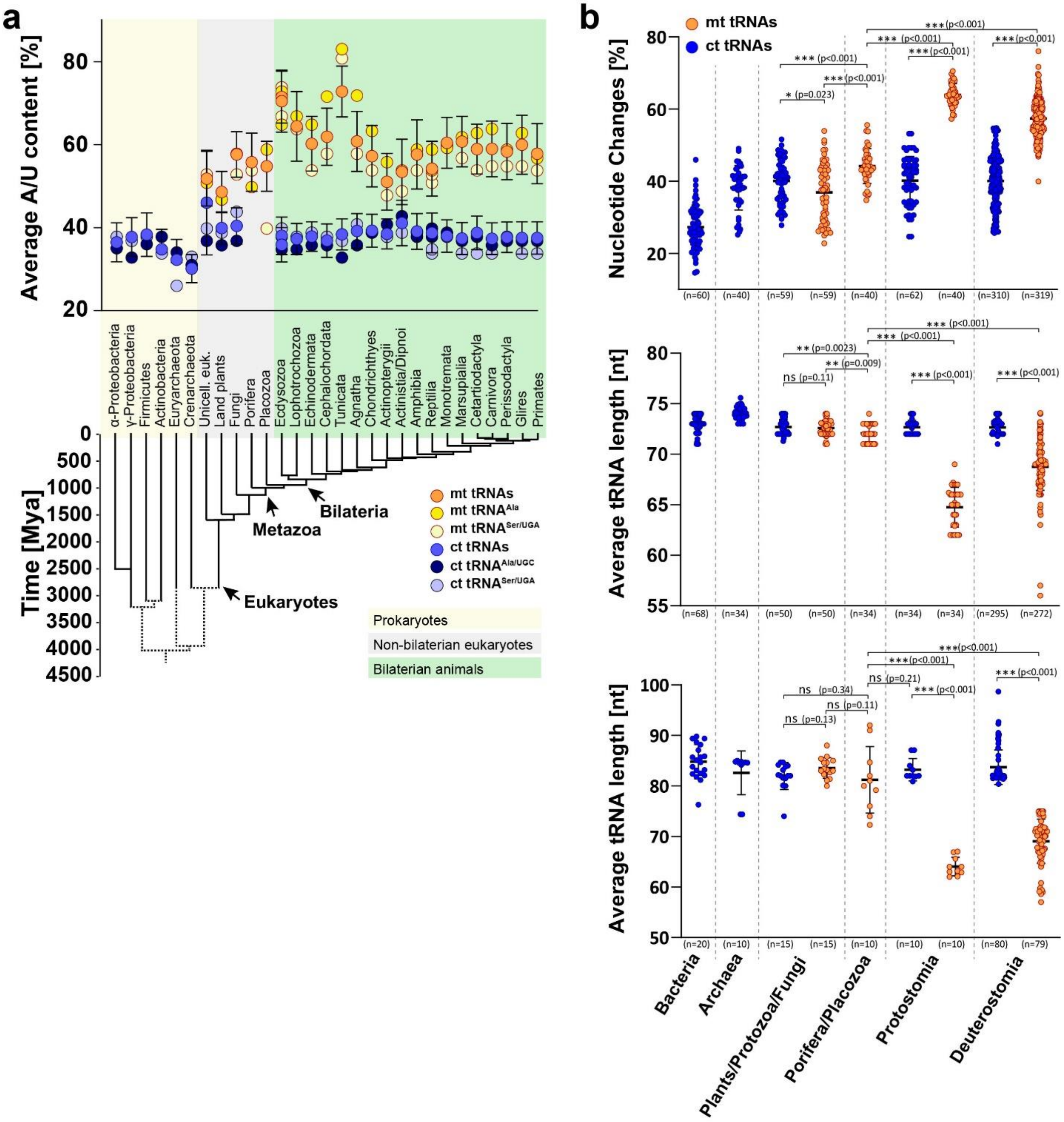

Supplementary Figure 1. a) A/U content in prokaryotic/cytoplasmic (ct) and mitochondrial (mt) tRNAs. For each phylogenetic lineage indicated below the x-axis, the average relative A/U content for prokaryotic/cytoplasmic (blue) or mitochondrial (orange) tRNAs was calculated. Dark and light yellow indicate individual average values for mt tRNA^{Ala} and mt tRNA$^{Ser/UGA}$, respectively; dark and light blue indicate individual average values for ct tRNA^{Ala} and ct tRNA$^{Ser/UGA}$, respectively. Prokaryotic/cytoplasmic tRNAs show a relative constant A/U content of around 40-43% throughout the analyzed phylogenetic lineages, with slightly lower content in archaeal tRNAs and a maximum of ~50% in unicellular eukaryotes. All mt tRNAs show increased A/U content

compared to their cytoplasmic counterparts, with the lowest percentages in unicellular eukaryotes (~56%) and land plants (~53%) and the highest in protostomes (68-75%), Echinoderms (~64%), Cephalochordates (~66%), and Tunicates (~77%). Interestingly, the A/U content is not elevated in most vertebrates compared to non-bilaterian metazoan mt tRNAs, with values of ~55% in Actinopterygii and 60-64% in Sarcopterygii. The pattern of increasing A/U content in mt tRNAs is similar to that observed for the accumulation of sequence changes in mt tRNAs (Figure 1a), suggesting that substitution of G:C with A:U is a major contributing factor. Error bars indicate standard deviations from the mean A/U content. **b)** Statistical analysis of data for average length and sequence changes of mitochondrial (orange) and prokaryotic/cytoplasmic (blue) tRNAs. Data shown in Figure 1a were grouped into nine subgroups: bacterial tRNAs, archaeal tRNAs, non-metazoan (land plants, unicellular eukaryotes/protozoa, fungi) ct tRNAs, non-metazoan mt tRNAs, non-bilaterian metazoan (porifera, placozoa) mt tRNAs, protostome ct tRNAs, protostome mt tRNAs, deuterostome ct tRNAs, and deuterostome mt tRNAs. Data for the sequence lengths of tRNAs was subdivided into class I tRNAs (with short V-loop; middle panel) and class II tRNAs (specific for Leu, Ser, Tyr; with long variable arm; bottom). Thick horizontal bars and error bars indicate mean values and standard deviations, respectively. Statistical significance ($p < 0.05$) or non-significance (ns) by the Wilcoxon rank sum test (two-tailed) is indicated for relevant pairs, with p-values indicated. Comparisons between the three eukaryotic ct tRNA groups show no significance in the three data sets (not indicated). $P < 0.001$ (***), $p < 0.01$ (**), $p < 0.05$ (*), $p > 0.05$ (ns). Source data are provided as a Source Data file.

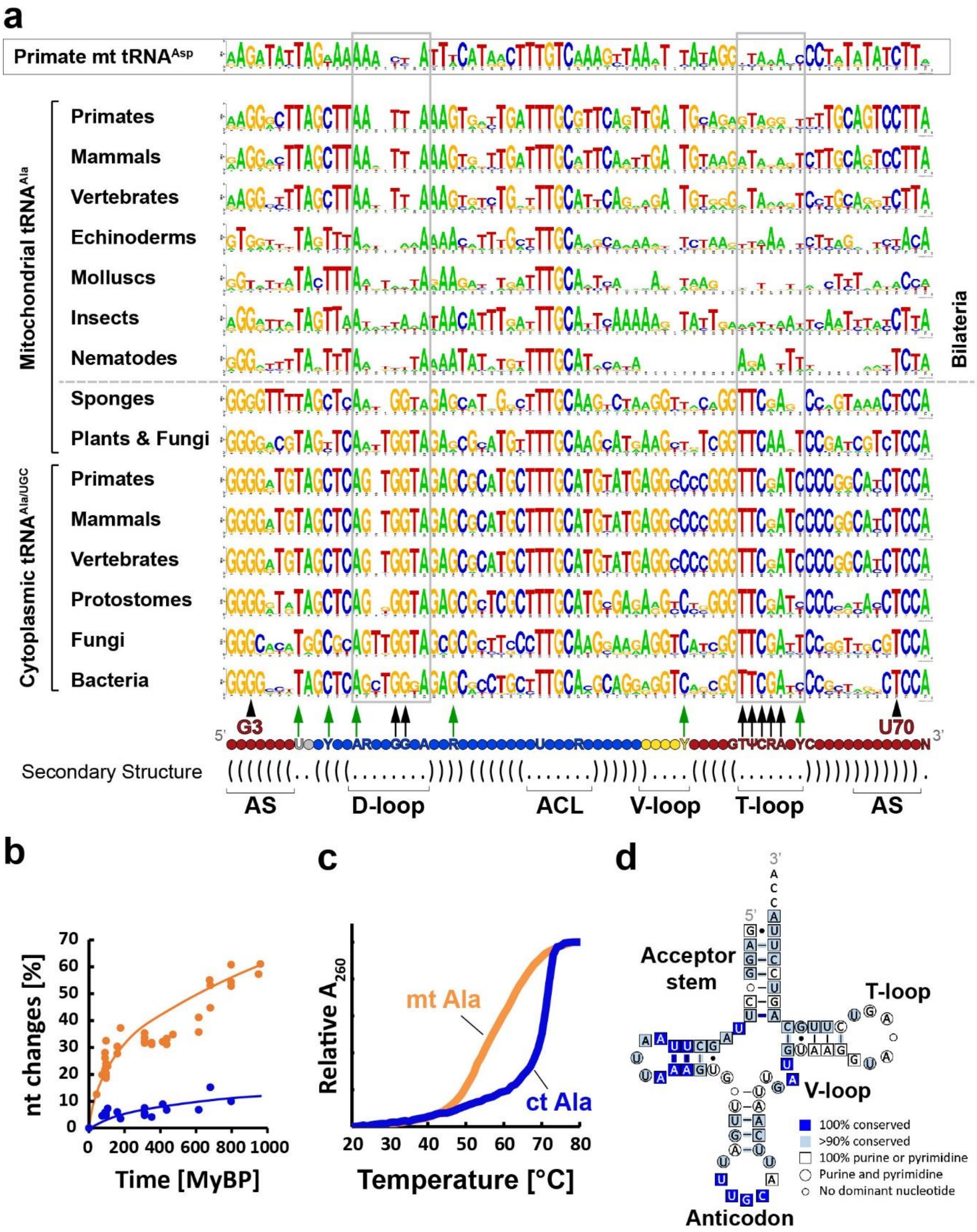

Supplementary Figure 2. a) Sequence logos showing divergence of tRNA$^{Ala/UGC}$ in mitochondria compared to prokaryotic and eukaryote cytoplasmic counterparts. Sequence logos were generated from alignments of tRNA^{Ala} from the phylogenetic groups indicated on the left. At the very top, the sequence logo for primate mt tRNA^{Asp} is shown for comparison. Information on the tRNA secondary structure is given at the bottom below the sequence logos, indicating the position of the acceptor stem (AS), D-loop, anticodon loop (ACL), variable loop (V-loop), and T-loop along the

primary sequence. Parentheses denote paired regions with '(' indicating the 5' side and ')' the 3' side of a stem; dots indicate unpaired regions. The color code and sequence information above the secondary structure plot is the same as in the cloverleaf presentation of canonical tRNAs in Figure 1b. 'G3' and 'U70' indicate the G3:U70 identity element in canonical $tRNA^{Ala}$ systems. Green arrows indicate structurally relevant positions that are at least partially conserved between canonical and mt $tRNA^{Ala}$ variants. Black arrows indicate structurally relevant positions that are conserved in canonical tRNAs but not conserved in bilaterian mt $tRNA^{Ala}$. Gray boxes frame the D- and T-loops. The gray dashed horizontal line indicates the transition between non-bilaterian and bilaterian animals. **b)** Nucleotide changes in mt $tRNA^{Ala}$ (orange) and ct $tRNA^{Ala/UGC}$ (blue) in bilaterian lineages relative to sequences from Old World monkeys. Values on the x-axis correspond to the divergence times relative to primates. The rapid increase in variable sites with increasing divergence time is specific to mt tRNAs, showing their high substitution rates compared to canonical tRNAs. **c)** Thermal melts of *in vitro* transcribed *Hs* mt $tRNA^{Ala}$ (orange) and ct $tRNA^{Ala}$ (blue). **d)** Sequence variability between mammalian mt $tRNA^{Ala}$. Shown is the cloverleaf presentation of mt $tRNA^{Ala}$ with a typical sequence, i.e. prevalent nucleotides among mammalian variants. Plotted onto the secondary structure is the conservation of each position according to the indicated color code. Only nine positions are invariant between mammalian mt $tRNA^{Ala}$ sequences (excluding the anticodon and 3'-CCA end). All nine positions are likely relevant for canonical core tertiary interactions. There is no invariant position within the acceptor stem. Related to Figure 1.

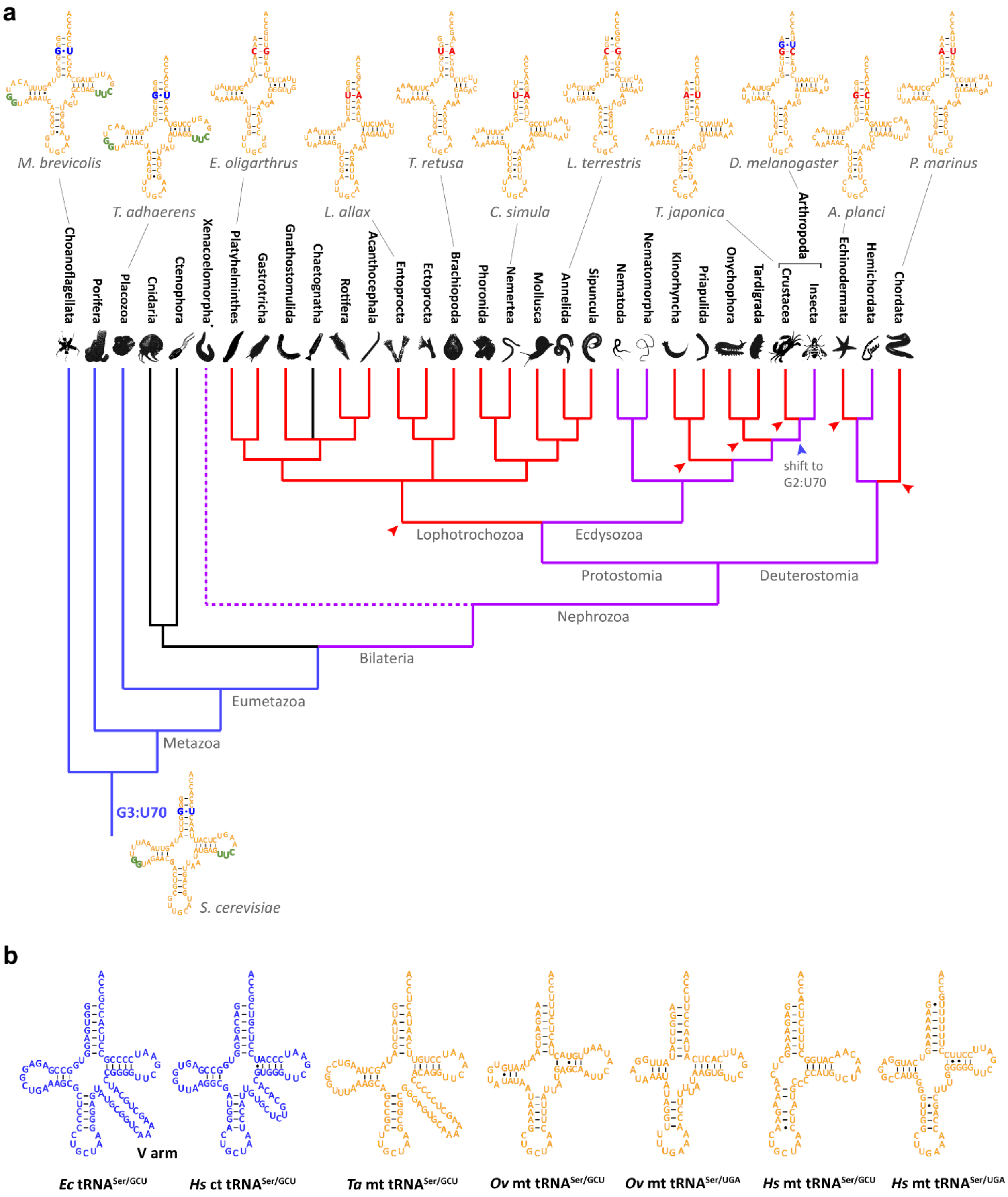

Supplementary Figure 3. a) Phylogenetic tree showing the distribution of the G3:U70 identity element in mt tRNA^{Ala}'s of major metazoan lineages. Blue lines indicate lineages in which mt tRNA^{Ala} has retained both, canonical structural features and the conserved G3:U70 identity element. Purple lines indicate lineages in which mt tRNA^{Ala} retain a conserved G3:U70 (or G2:U70 in the case of insects) but have degenerated structural features in the D- and T-loops. Red lines indicate lineages in which G3:U70 is lost as a conserved element in extant mt tRNA^{Ala} variants

together with the degeneration of canonical structural features. Red arrows indicate possible events of loss of G3:U70 from mt tRNA^{Ala}, which suggest that it was lost at least two times independently, once in protostomes and once in deuterostomes. Black lines indicate lineages for which mitochondrial genomes were sequenced, but which do not encode a tRNA^{Ala} gene (not shown are major bilaterian lineages without sequenced mtDNA genomes). At the base of the tree, the mt tRNA^{Ala} from *S. cerevisiae* is shown, with G3:U70 indicated in blue and G18, G19 in the D-loop and the UUC trinucleotide in the T-loop highlighted in green. Above the tree, examples of mt tRNA^{Ala}'s from various different phyla are shown, with nucleotides in lieu of G3:U70 highlighted in red. The phylogenetic relationships of metazoan phyla are based on information from Timetree (http://www.timetree.org/) and references therein and do not contain temporal information. *The placement of Xenacoelomorphs is not clear, as they were classified either as a basal bilaterian sister group to Nephrozoa or as a basal deuterostome; the unclear placement is indicated by dashed lines. It should be noted that neither a purple line, nor a red line indicates 100% presence or 100% loss of G:U wobble pairs from the lineage. G:U wobble pairs occur repeatedly in acceptor stems throughout bilaterian mt tRNA^{Ala} variants (and other mt tRNA specificities), but are usually not conserved even between closely related species. On the one hand for example, mt tRNA^{Ala} from *Eremitalpa granti* (Grant's golden mole) contains a G3:U70 pair, while the closely related *Chrysochloris asiatica* (Cape golden mole) contains G3:C70. Similarly, *Albinaria caerulea* is the only mollusk known to have G3:U70 in its mt tRNA^{Ala}, suggesting that it represents a neutral transitional state from the more common A3:U70. At the same time, G3:U70 is common in multiple other mollusk mt tRNA specificities such as mt tRNA^{Arg} and mt tRNA^{Ser}. On the other hand, bilaterian lineages that are indicated as having retained the G3:U70 (purple lines) may also contain mt tRNA^{Ala} genes that do not encode G3:U70 in a minority of species. For example, mt tRNA^{Ala} in three Hemichordate species contain G3:U70, while that of *Saccoglossus kowalevskii* does not (G3:C70 instead). **b)** Examples of tRNA^{Ser} isodecoders from *E. coli* (*Ec*), human (*Hs*), *Trichoplax adhaerens* (*Ta*), and *Octopus vulgaris* (*Ov*). Bacterial and eukaryote cytoplasmic tRNAs are shown in blue, mitochondrial tRNAs in orange. Notably, the long variable arm that is used as identity element in canonical recognition systems is present in the placozoan *T. adhaerens* but is lost in both isodecoders of protostome and deuterostome species. Related to Figure 1.

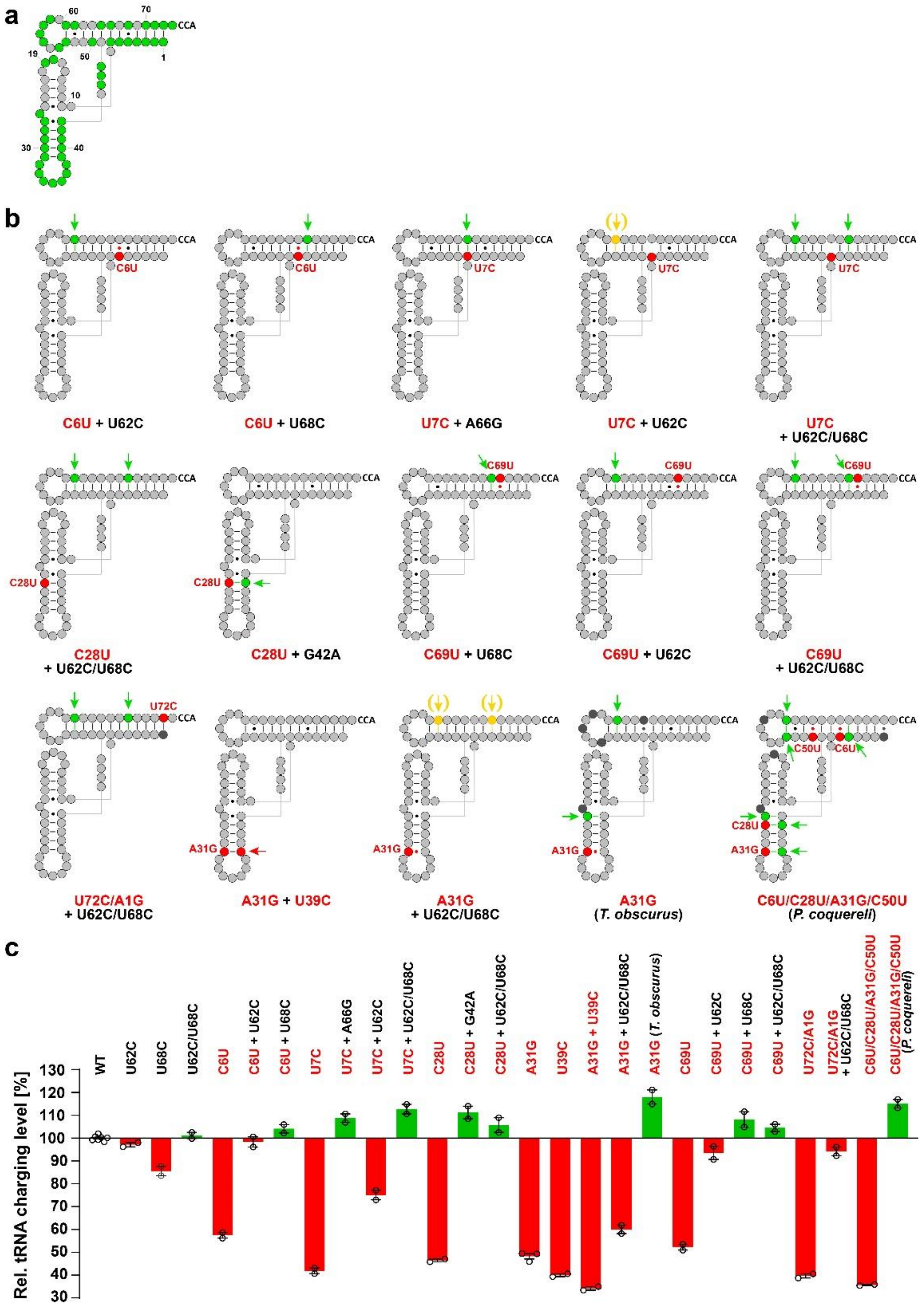

Supplementary Figure 4. Compensatory epistasis in *Hs* mt tRNA^{Ala}. **a)** Schematic presentation of the L-shaped *Hs* mt tRNA^{Ala}. Highlighted in green are all positions that were mutated in this study that had no apparent effect on charging activity by *Hs* mt AlaRS. Some of the positions had a negative effect when mutated individually but showed no effect if in combination with compensatory mutations. Related to Figures 2 and 3. **b)** *Hs* mt tRNA^{Ala} is presented in schematic

L-shape as in a). Individual constructs used in this study show compensatory epistasis between sites. Red indicates initial mutations that reduce charging. The positions for compensatory mutations are shown in yellow (partial compensation) and green (complete compensation) and are indicated by arrows. Black circles indicate positions with sequence alterations relative to wild-type *Hs* mt $tRNA^{Ala}$ with unknown effects on stability. **c**) Relative charging levels of the various tRNA constructs. Interestingly, the U72C mutation – at the end of the acceptor stem – was not compensated by A1G, even though it introduced a more stable Watson-Crick pair. Yet, combining A1G/U72C with the distant U62C/U68C mutations restored activity. Similarly, the A31G mutation – introducing a G31:U39 wobble pair at the base of the anticodon stem – was not compensated by U39C. Secondary structure prediction analysis suggested that the A31G substitution promoted alternative folding into an extended hairpin that is further reinforced by C39 (see also Supplementary Figure 5d/e). The charging level of wild-type (WT) *Hs* mt $tRNA^{Ala}$ is set to 100%. Error bars represent the SEM of two to seven independent experiments. Source data are provided as a Source Data file. (Related to Figure 3 and Table 2)

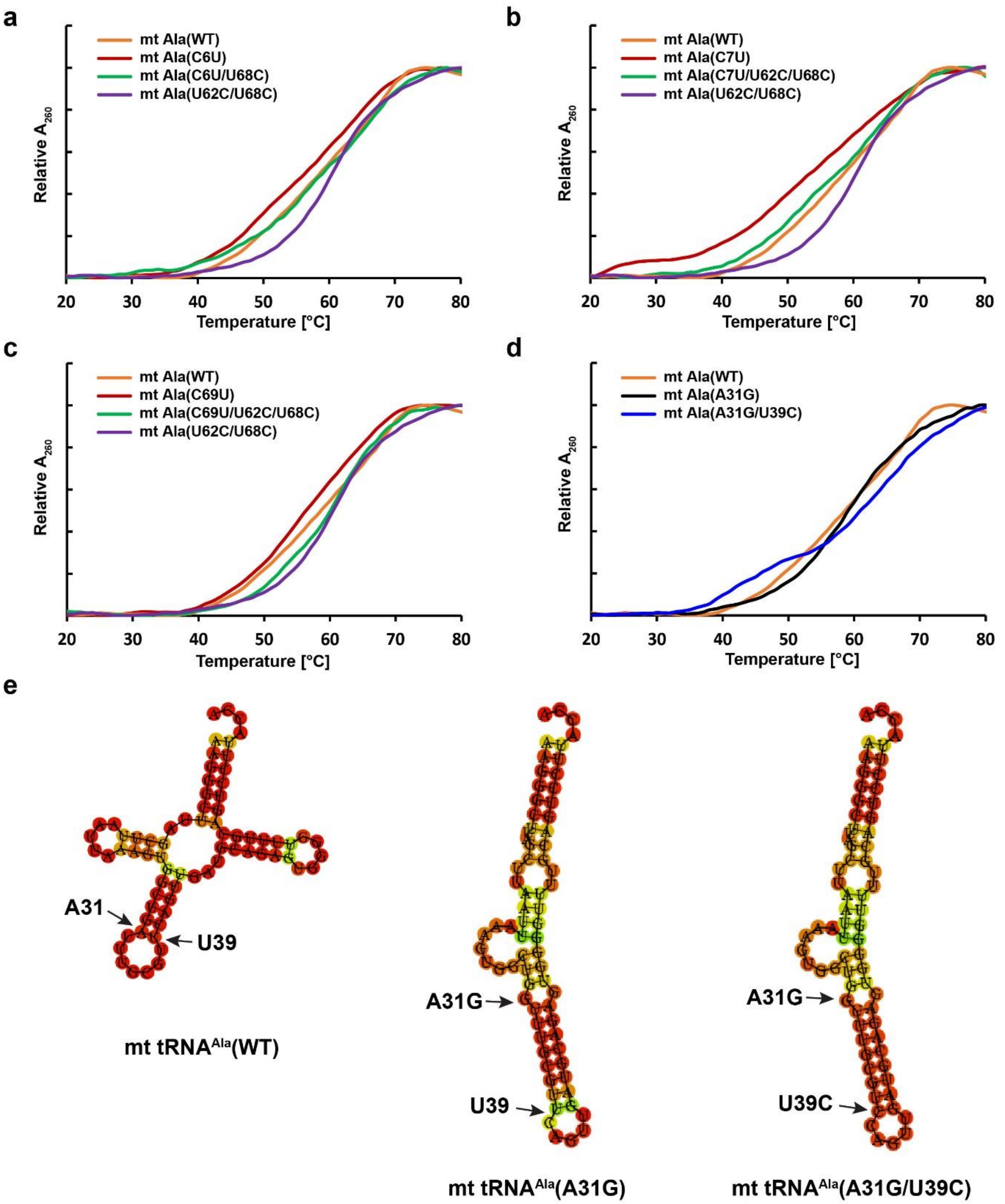

Supplementary Figure 5. a-d) Thermal melting curves for mt $tRNA^{Ala}$ variants used in Fig. 3 and Supplementary Figure 4. **e)** Secondary structure predictions for wild-type (WT) mt $tRNA^{Ala}$ (left), the single site mutant A31G (middle) and the A31G/U39C double mutant (right). Although A31G/U39C introduce a more stable G:C base pair instead of A:U into the anticodon stem of the native fold, it also stabilizes an alternative misfolded state (right), possibly explaining the loss of charging activity by mt AlaRS (see Supplementary Figure 4) despite increased thermal stability of the RNA fold. Predictions were done using the ViennaRNA Package (https://www.tbi.univie.ac.at/RNA/). Related to Figure 3.

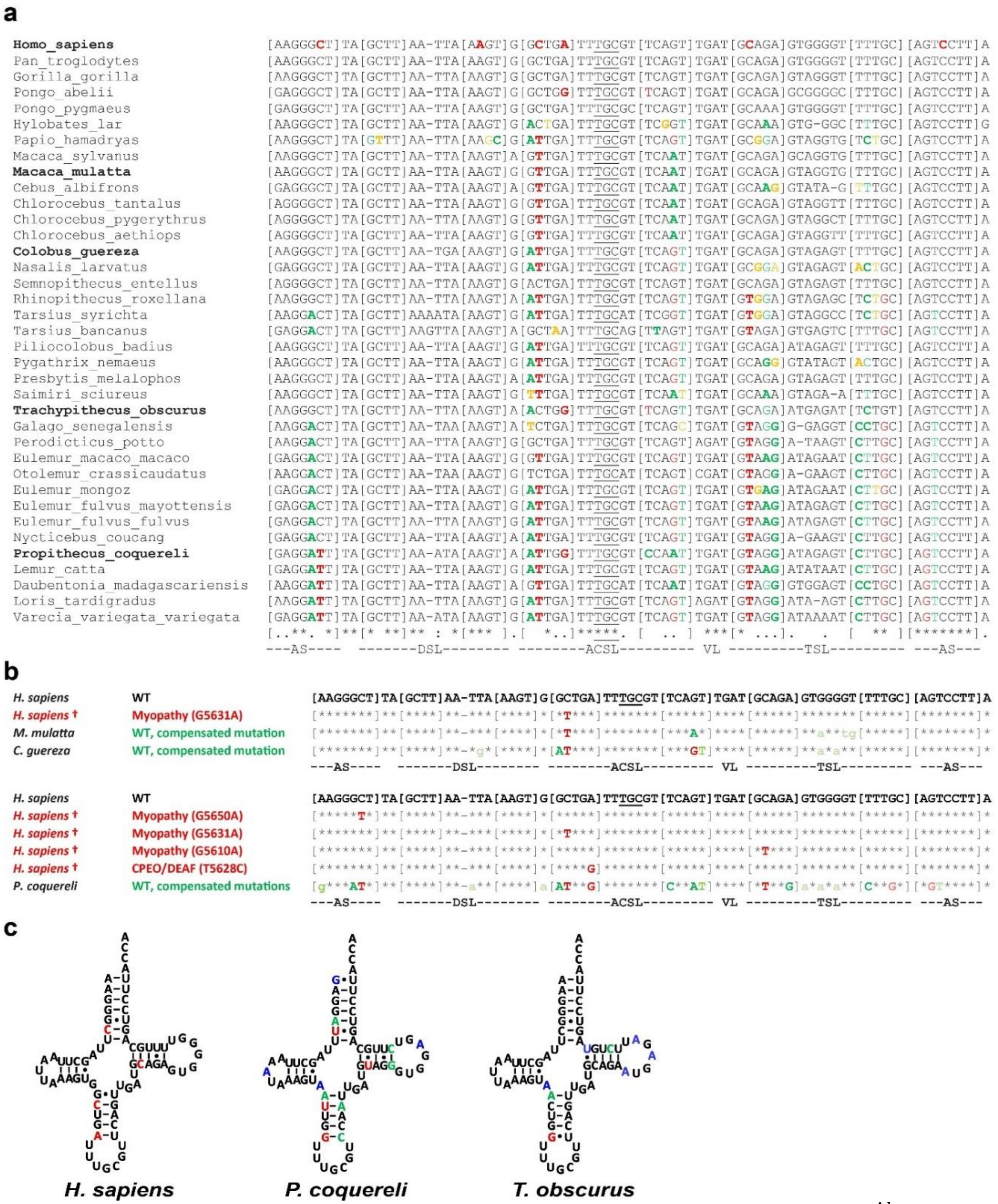

Supplementary Figure 6. a) Multiple sequence alignment of primate mt tRNA^{Ala} sequences with compensated pathogenic sites. Bold red indicates sites of disease-causing mutations reported for human mt tRNA^{Ala} and corresponding sites mutated in primate mt tRNA^{Ala}. Sites base-pairing to mutated sites are in red. Presumed compensating mutations are shown in green. Alterations in primate mt tRNA^{Ala} that likely destabilize the secondary structure relative to the human tRNA are shown in yellow. **b-c)** Specific examples of compensated pathogenic sites in primate mt tRNA^{Ala}. Asterisks in b) indicate positions that are identical to the human sequence. In c) blue indicates positions that differ from human mt tRNA^{Ala} without obvious effect on stability.

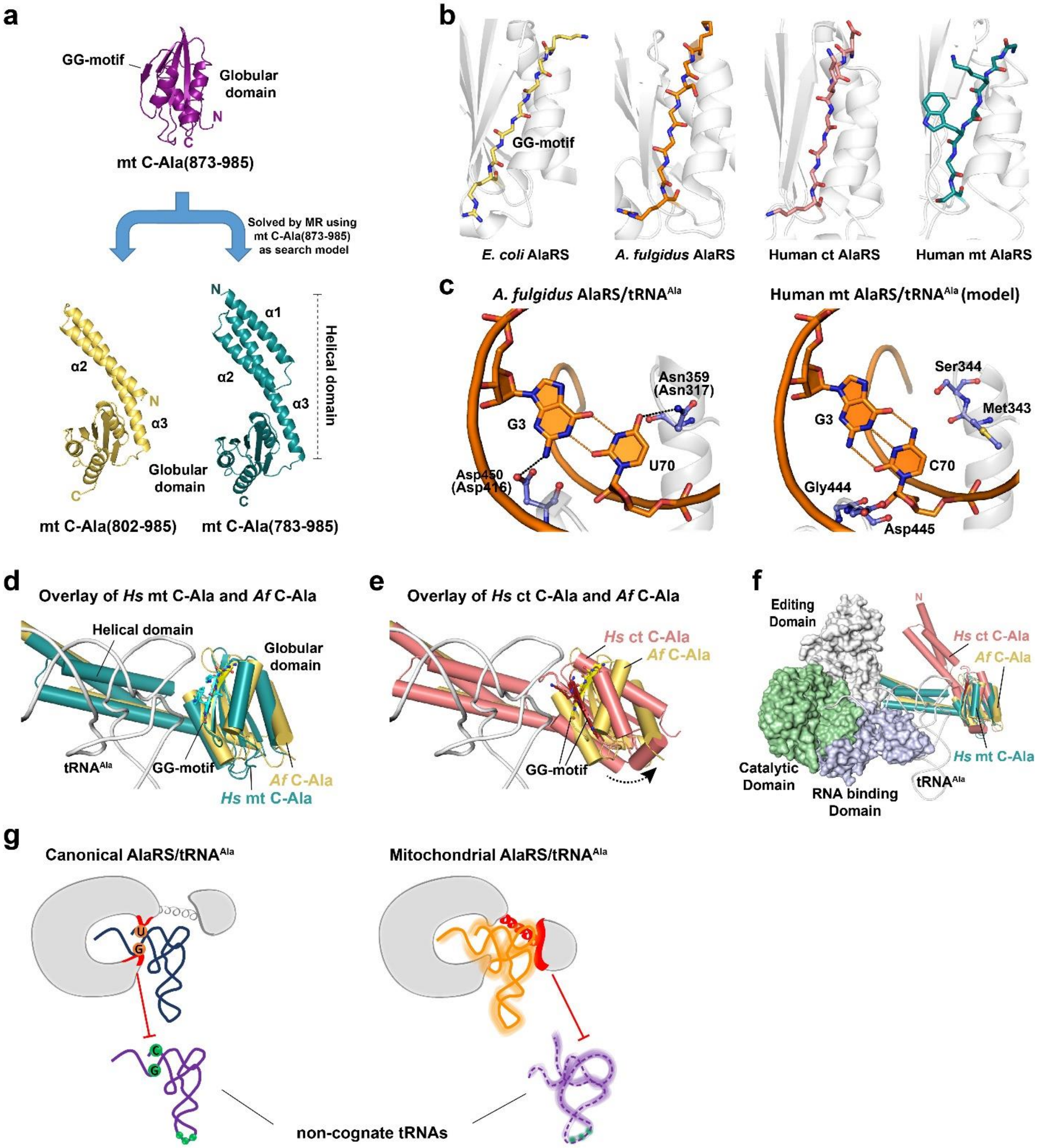

Supplementary Figure 7. a) Crystal structures of the C-terminal domain (C-Ala) from human mt AlaRS solved in this study. The C-terminal globular domain (aa 873-985) was solved by molecular replacement using the previously solved structure of cytoplasmic C-Ala (C-terminal domain; PDB: 5T76) as search model. C-Ala(873-985) was used as search model to solve the structures of mt C-Ala(802-985) and C-Ala(783-985). **b)** Detail of the GG-motif (shown as sticks) in C-Ala structures from canonical alanine recognition systems (*E. coli*, *A. fulgidus*, and human cytoplasm) compared to the altered 'GG-motif' found in human mt AlaRS, in which a Trp residue is inserted into the otherwise conserved stretch of glycines. **c)** Left: Detail of the conserved recognition dyad of Asn

and Asp interacting with G3:U70 in the acceptor stem of canonical tRNA^{Ala} (PDB: 3WQY). Positions in *Hs* ct AlaRS are given in parentheses. Right: In *Hs* mt AlaRS, the recognition dyad is lost and replaced by Met343 (for Asn317) and Gly444 (for Asp450). Instead of G3:U70, Hs mt tRNA^{Ala} contains a G3:C70 base pair which acts as anti-determinant in the canonical recognition system. **d-e)** Structure comparison of human mt C-Ala (cyan) and human ct C-Ala (salmon) with *A. fulgidus* C-Ala (yellow) in the context of the *A. fulgidus* AlaRS/tRNA^{Ala} complex. C-Ala structures were superimposed based on the helical N-terminal subdomain. While the globular domain of human mt C-Ala is arranged in the same way as that of *A. fulgidus* C-Ala and is thus able to interact with tRNA (gray tube) in the same manner using the GG-motif, the globular domain of human ct C-Ala is rotated relative to the helical subdomain (arrow) and thus does not allow similar interactions with the tRNA. **f)** Superposition of human mt C-Ala, ct C-Ala and *A. fulgidus* C-Ala (colors as in d and e) based on the globular subdomain. The N-terminus of ct C-Ala points away from the C-terminus of the editing domain, showing that the orientation of its globular subdomain relative to the N-terminal helical subdomain is incompatible with tRNA binding in the AlaRS/tRNA complex. **g)** Scheme highlighting the differences between canonical alanine recognition systems conserved from prokaryotes to the human cytoplasm (left), and the human mitochondrial system (right). During evolution the recognition mechanism changed from sequence-specific readout of identity elements (G3:U70 for alanine) embedded in the universal scaffold of canonical tRNAs to a sequence-unspecific structure-based readout of the tRNA elbow/core. Rejection of non-cognate mt tRNAs in the mitochondrial system is based on structural incompatibility rather than the presence or absence of specific sequence elements. Red indicates surfaces on AlaRSs that are responsible for cognate tRNA recognition. Related to Figure 4.

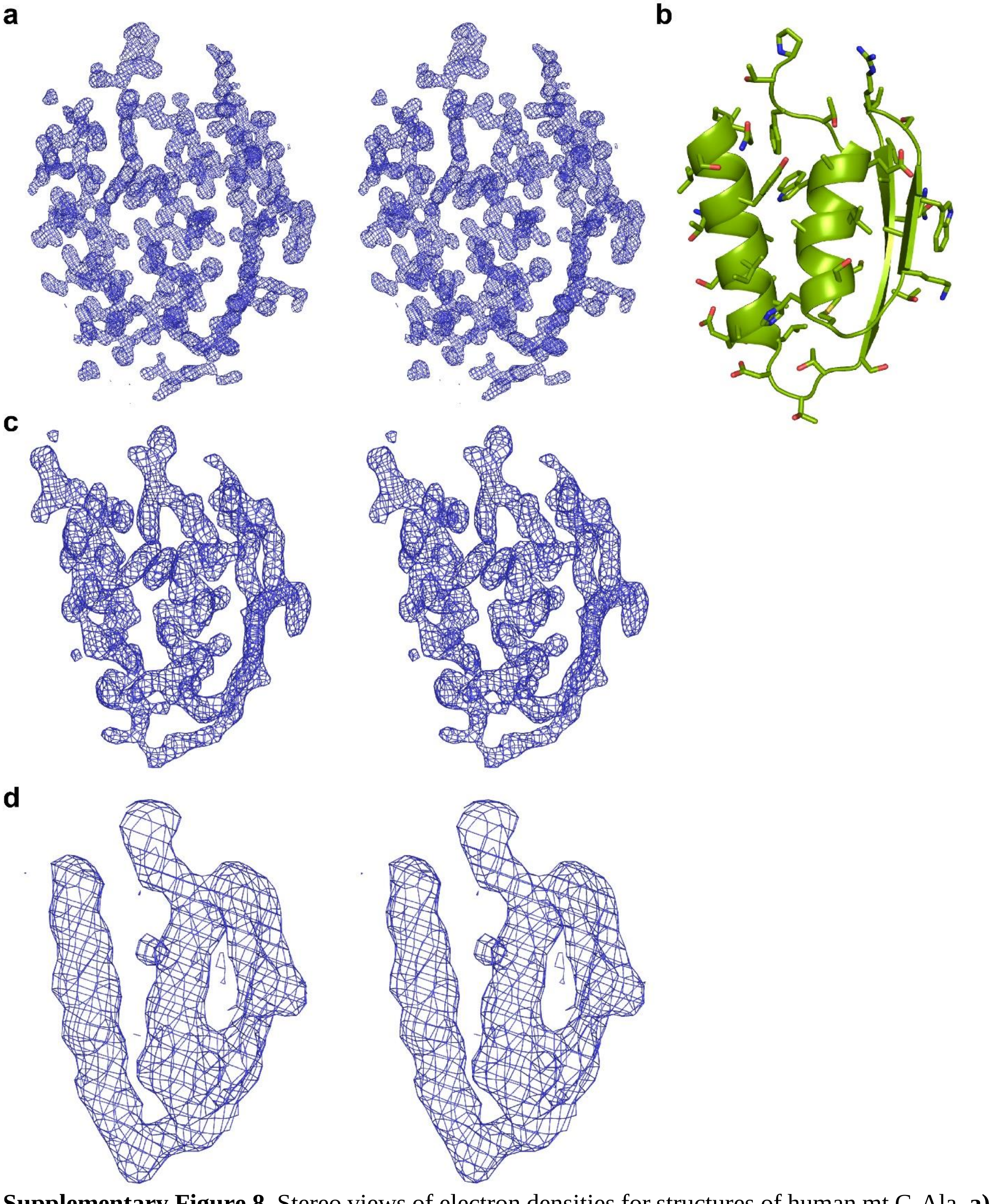

Supplementary Figure 8. Stereo views of electron densities for structures of human mt C-Ala. **a)** Stereo view of the 2Fo-Fc electron density map for residues 935-985 in C-Ala(873-985) contoured at 1.5σ. **b)** Cartoon and stick presentation of the partial structure of C-Ala (residues 935-985) for which electron densities are shown in panels a, c, and d. **c)** Stereo view of the 2Fo-Fc electron density map for residues 935-985 in C-Ala(802-985) contoured at 1.5σ. **d)** Stereo view of the 2Fo-Fc electron density map for residues 935-985 in C-Ala(802-985) contoured at 1.2σ.

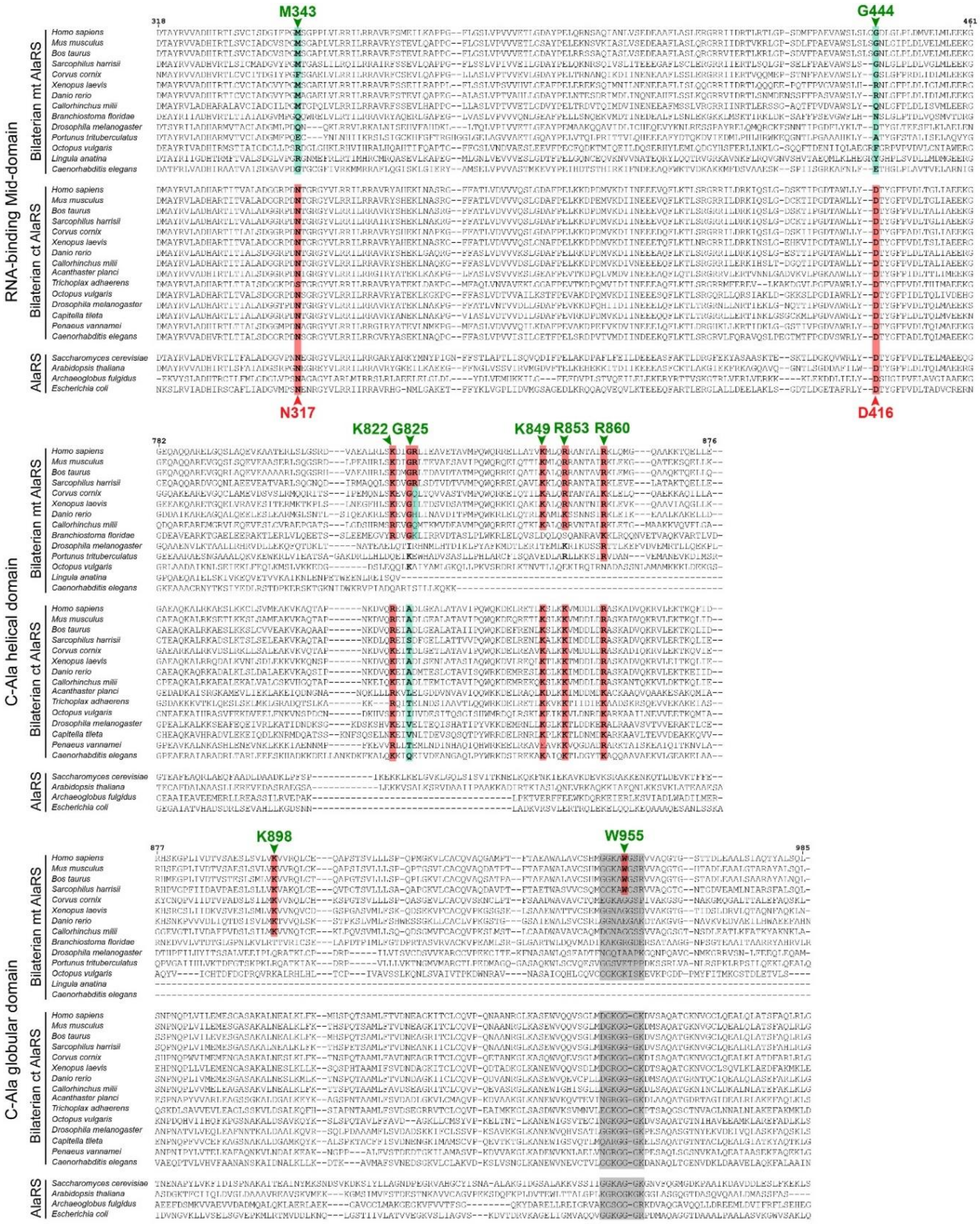

Supplementary Figure 9. Multiple sequence alignment of AlaRS in the RNA-binding domain (top), the helical N-terminal subdomain of C-Ala (middle) and the globular C-terminal subdomain of C-Ala (bottom). The GG-motif is highlighted by gray background. N317 and D416 correspond to the recognition dyad in *Hs* ct AlaRS, which is conserved in cytoplasmic AlaRSs but replaced by other residues in mitochondrial AlaRSs. Note that non-bilaterian AlaRSs are dual localized, functioning both as cytoplasmic and mitochondrial synthetases e.g. in *S. cerevisiae* and in *A. thaliana*. Related to Figure 4.

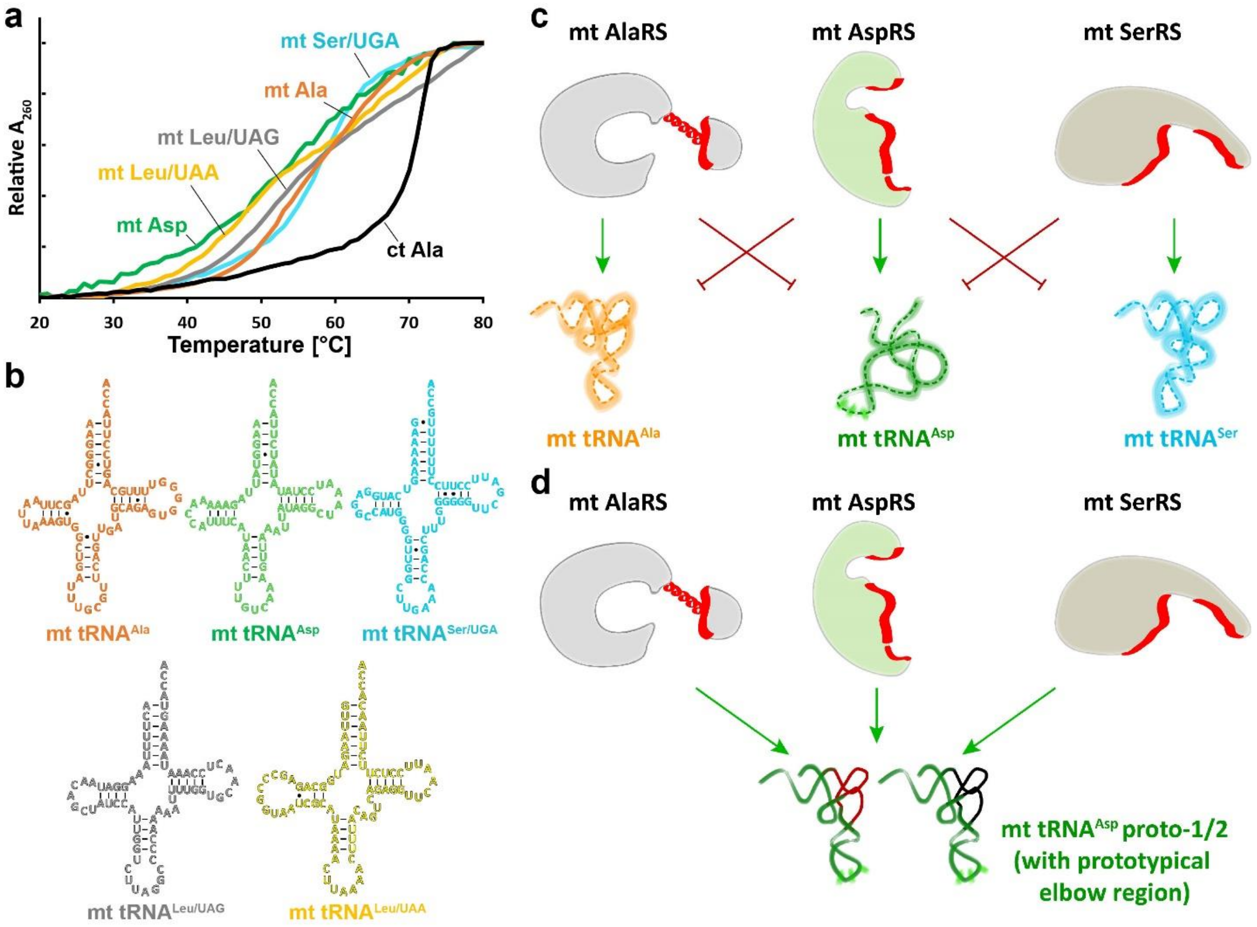

Supplementary Figure 10. a) Thermal melts for human mt tRNAs used in this study and ct tRNA^{Ala}. **b)** Cloverleaf presentation of the mt tRNAs used in this study. **c)** Schematic presentation of the human mitochondrial recognition system. Structural flexibility and incompatibility serve as anti-determinants between non-cognate systems. Mt aaRSs selectively provide stability to their cognate tRNA substrates, while rejecting structurally incompatible non-cognate tRNAs. **d)** The discrimination barrier can be overcome by the introducing prototypical elbow regions into an unstable mt tRNA, thereby introducing increased stability and bypassing the requirement for stabilization by the aaRS. Related to Figure 5.

Supplementary Table 1.
Data collection and refinement statistics. Related to Figure 4.

**Data Collection**	**mt C-Ala(873-985)**	**mt C-Ala(802-985)**	**mt C-Ala(783-985)**
PDB-ID	6NLQ	6NLY	6NOW
Space group	$P2_1$	$C222_1$	$I4_1$
Cell dimensions			
a, b, c (Å)	55.19, 66.95, 56.48	103.22, 317.23, 54.71	104.42, 104.42, 164.27
α, β, γ (°)	90.00, 116.42, 90.00	90.00, 90.00, 90.00	90.00, 90.00, 90.00
Resolution (Å)	38.71–1.15 (1.22–1.15)	38.45–2.31 (2.45–2.31)	35.89–4.09 (4.34–4.09)
R_{meas} (%)	4.5 (16.9)	5.0 (64.4)	6.0 (53.0)
I/σ(I)	14.12 (5.0)	17.7 (2.8)	17.47 (3.49)
$CC_{1/2}$	99.6 (96.6)	100.0 (92.0)	99.9 (96.2)
Completeness (%)	91.3 (81.1)	99.2 (98.9)	99.7 (99.4)
Redundancy	1.7 (1.6)	3.8 (3.9)	7.6 (7.5)
**Refinement**			
Resolution (Å)	38.71–1.15	38.02–2.31	35.89–4.09
No. of reflections	123977	39915	6884
R_{work} (%)	15.85	20.94	23.32
R_{free} (%)	17.23	25.70	28.20
No. of atoms			
Protein	3424	5400	2914
Water	500	17	0
Ions	3	0	0
B-factor (Å^2)	18.1	77.4	259.8
r.m.s. deviations			
Bond lengths (Å)	0.012	0.011	0.003
Bond angles (°)	1.461	1.415	0.653
Ramachandran plot			
favored (%)	98.5	98.9	98.0
outliers (%)	0.0	0.0	0.0
Rotamer outliers (%)	1.0	0.9	0.0
Clash score	8.82	11.85	9.8

Values in parentheses refer to the highest resolution shell. Each structure was determined from one crystal.
