# Supplementary material for: Relaxed sequence constraints favor mutational freedom in idiosyncratic metazoan mitochondrial tRNAs
Source: Nat Commun. 2020 Feb 20;11:969. doi: 10.1038/s41467-020-14725-y (PMC7033119; doi:10.1038/s41467-020-14725-y)
Supplement: Supplementary file 2 — Peer Review [file 41467_2020_14725_MOESM2_ESM.pdf]

Reviewers' comments:

Reviewer #1 (Remarks to the Author):

The manuscript deals with the aspect of how mitochondrial translation (tRNA and aaRS) system adapts to the high mutational rate of the mitochondrial genome. Further, the authors have performed bioinformatic and biochemical studies based on which they claim that a G3:U70-independent recognition of tRNAAla is facilitated by key adaptations at the mitochondrial AlaRS level. Interestingly, they show that the aminoacylation barrier can be overcome by transplanting the variable arm (in particular the elbow region) thus identifying a mitochondrion specific tRNA identity element for tRNAAla system.
Overall, the manuscript is trying to link the above experimental observations to a larger phenomenon of mutational freedom in mt tRNAs which is possibly linked to the evolution of bilaterians. In my opinion, the claim is too tall and not sufficiently supported (see below). Also, the authors choose systems and examples arbitrarily to make their point while conveniently leaving out the exceptions!

Major concerns:
a) One of the major claims of the article is 'bilaterians mt tRNA have lost the identity determinants and are unstable'.
• The unusual size and structure of mt tRNAs were previously noted (Suzuki et al, Annual Review of Genetics 2011, 45:1, 299-329; Salinas-Giege et al, Int. J. Mol. Sci. 2015, 15, 4518-4559).
• Is there any rationale to analyse only 9 out of 20 amino acid-specific tRNAs? Supplementary Figure 1 includes tRNAs only from primates and mammals. What is the scenario with respect to lower bilaterians and non-bilaterians mt tRNAs?
• The loss of identity determinants in mt tRNAAla for AlaRS charging was reported in previous studies and an alternate mode of recognition was proposed. The G3:U70-independent mode of recognition by mt AlaRS was beautifully shown very recently by En-Duo Wang and co-workers, wherein multiple positions on the tRNA are recognized by the mitochondrial AlaRS (Zeng et al, Nucleic Acid Research 2019, 47:6, 3072-3085).
• The authors claim that G3:U70 is entirely lost from the metazoans mitochondria. In contradiction to this, it has been previously (Chihade et al, Biochemistry 1998, 37:25, 9193-9202) shown that C. elegans mitochondrial AlaRS uses G3:U70 as a determinant for charging the mt-tRNAAla. Like C. elegans, other bilaterians such as Albinaria caerulea and Ascaris suum also have a G3:U70 in the mt-tRNAAla. Hence the metazoan/bilaterian based argument does not hold good and unfortunately the authors neither choose to point these out or provide an explanation for these violations.
• Previous work from the same group has shown that Drosophila melanogaster mtAlaRS recognizes the translocated G2:U71 of mt tRNAAla, while G3:U70 acts as a negative determinant for aminoacylation (Lovato et al, EMBO 2001, 20:4846-4853). Hence, the universality of G:U recognition in tRNAAla was put forward using the above data from the mitochondrial system as well. In light of these studies, the argument of bilaterian specific phenomenon becomes fragile and needs to be revisited.

b) The second major claim is the 'molecular adaptation of mtAlaRS to recognize the elbow region of the tRNA by creating a positive patch in the C-Ala domain, which is essential for aminoacylation'.
• The mutational analysis of the C-Ala domain (positive patch) shows that K822, R853, R860 are important for recognizing the tRNA. However, these residues are expected to be specific to mtAlaRS only, but (from supplementary figure 5) these residues are also conserved in ctAlaRSs. Thus, the claim for the appearance of the positive patch only in C-Ala domain of bilaterian mtAlaRS is questionable!
• R826 of mtAlaRS is not conserved but mutation to Alanine does affect the aminoacylation rate in one case. Does that mean that the proposed model is specific to only a very small subset of higher organisms?
• The authors present the sequence alignment of AlaRS from different organisms. However, for mtAlaRS the list includes only chordates while leaving out the lower bilaterians/metazoans.

• As the structure solved here is only the C-Ala domain and the interpretation is based on the full-length model stating "In particular, the mt AlaRS-specific Gly825 allows tight association between C-Ala and tRNA" may not be appropriate. Do the authors mean that introducing G825 (along with M343, G444) in ctAlaRS is sufficient enough to charge mt-tRNAAla?

c) The authors generalize the mechanism of "chaperon-like fitting" to the rest of the mt synthetases.
• A major premise of this manuscript is "We hypothesized that, in contrast to the rigid lock-key selection of cytoplasmic AARS-tRNA pairs, an adaptive chaperone-like fitting of the nuclear encoded synthetase with its mt tRNA partner made possible the mutational freedom of mt tRNAs". Due to the multidomain architecture of aaRSs, they are shown to be involved in an induced-fit mode of enzyme-substrate interaction (Sherlin & Perona, Structure 2003, 11: 591–603). The induced fit/conformational selection of tRNA-synthetase recognition is so well known in the field and therefore it is difficult to imagine that the authors choose to present a totally different narrative!
• In abstract the authors state "Outsourcing structural stability to nuclear-encoded aaRS partners" which is incorrect. By using the term "Outsourcing" does the authors mean the stability of mt aaRS is more compared to ct aaRS? Since the complex possibly is more stable, there is no 'outsourcing' happening here!
• As correctly quoted (line 208), earlier studies have shown that mtSerRS uses tRNA elbow region for its charging. As the entire experimental proof for the current hypothesis is based on AlaRS and SerRS which do not follow the usual anticodon based tRNA recognition, any extrapolation or generalization to the rest of the mt synthetases may not be appropriate.
• Earlier studies have shown that in case of mitochondria the number of determinants for aminoacylation are reduced but not rewired fully (Suzuki et al, Annual Review of Genetics 2011, 45:1, 299-329).

d) tRNA sequence-independent recognition of mt tRNAAla by mt AlaRS.
• In line 137, the authors contradict the recent finding (Zeng et al, Nucleic Acid Research 2019, 47:6) and state that the tRNA recognition by mtAlaRS is based on the structure but not the sequence of the mt tRNAAla. Given the inadequate structural and biochemical data available, it is premature to say that tRNA structure plays an important role but not sequence. Perhaps, it appears that it is the combination of both which is recognized by mtAlaRS. In any case, the current work does not go beyond what has been published by Zeng et al. NAR 2019 in this aspect.

Other concerns:
• Line 23: 'aminoacyl tRNA' should be written as 'aminoacyl-tRNA' in the abstract.
• Line 26: 'Or' needs to be replaced by 'Our' in the abstract.
• Line 136: The non-reverting mutation of mt tRNAAla U54/58 is not shown in Figure 3c or table1-3.
• In Figure 5c the activity is plotted relative to tRNAAsp, instead, these values need to be compared with mt tRNAAla.
• Line 210: The authors do not include Ser in the earlier bioinformatics analysis (Line 70), but use them as a proof of concept.
• Here the authors use SerRS as a proof of concept. Is it possible to show with other more canonical mt synthetases?
• Line 502: The reference volume and page numbers are incorrect.
• Table 2: the tRNA mutation 'G1:C73/C62/C68' is incorrect, and should be changed to 'G1:C72/C62/C68'

Reviewer #2 (Remarks to the Author):

This important manuscript describes new phenomena relevant to understanding how the emergence of mitochondria as an efficient energy source impacted the evolutionary emergence of higher-order animals with bilateral symmetry. The notional steps in the argument are these: (i) mitochondria

reproduce asexually and without recombination, making them potentially vulnerable to irreversible accumulation of deleterious mutations (i.e. Muller's ratchet); (ii) mitochondrial transfer RNAs exhibit extraordinary variations in size and sequence, compared to the more stable cytoplasmic tRNAs; (iii) mitochondrial tRNA variants are both structurally less complex and relatively unstable; (iv) the cognate mitochondrial aminoacyl-tRNA synthetase structures—encoded in the nucleus—compensate these vulnerabilities by developing unusual induced-fit binding mechanisms quite distinct from those of the cytoplasmic cognate pairs. As each of these statements opens substantial questions, the manuscript is necessarily highly speculative. Nonetheless, the authors do a credible job shoring up each conclusion.

The manuscript combines several diverse threads from the Schimmel laboratory, including their interest in disease-causing mutations mitochondrial tRNAs, significant markers of the major branch points in the evolution of higher organisms, and their long-standing contributions to the structural enzymology of aminoacyl-tRNA synthetases. The paper should be highly interesting to a broad spectrum of readers.

The work described was motivated by detailed studies of the disease-causing mutations in the mitochondrial tRNAAla. The salient conclusion from an impressive number of observations and related experiments in this manuscript is that these disease-causing mutations can be compensated by mutations at other sites (epistasis) as well as by transformed binding interfaces with the cognate synthetase. In particular, chimeric tRNAAla molecules in which regions of the cytoplasmic tRNAAla are replaced by their counterparts from mitochondrial tRNAAla restored the ability to acylate the cytoplasmic chimeras. Because these experiments suggested that context, rather than sequence determined binding, an induced-fit binding mechanism involving the elbow region appears critical for productive binding.

The authors describe structural data pertinent to this phenomenon by solving the domain responsible for binding the elbow from the mitochondrial synthetase, and combine it with artful homology modeling to show the extensive co-adaptation that shifted the recognition mechanism from a highly focused and sequence-dependent recognition of the (G3:U70) base pair, used almost universally by AlaRSs, to a more sequence-independent recognition of the tRNA elbow in the human mitochondrial AlaRS•tRNAAla complex.

Similar, but more limited analysis of mitochondrial tRNASer reveals comparable changes, suggesting that the mitochondrial alanine system is representative of a detailed distinction between how tRNAs are recognized by cytoplasmic aminoacyl-tRNA synthetases and (nuclear encoded) mitochondrial synthetases. An imaginative speculation by the authors is that the nuclear synthetase genes for mitochondrial synthetases co-adapt to the rapid sequence evolution of mitochondrial tRNA synthetase genes by developing chaperone-like induced-fit recognition mechanisms that preserve the coding specificity necessary to ensure survival of the bilateran energy-generating mitochondria, whose genomic DNA must evolve under higher oxygen tension.

The paper is quite well-written and conclusions are fully documented. The authors might consider the following suggestions:

1. P. 2, line 26. "Or" should probably be "Our".
2. P. 2, line 27. "evolution of animal complexity". Are there important differences between the bilateran mt tRNA sequences and those from the plastids of other higher organisms (i.e. chloroplasts).
3. P. 3, lines 59-62. The authors run the risk of making to sharp a distinction here between "lock and key" and induced-fit. Most of the cytoplasmic synthetases are likely to exhibit induced fit in various ways as well, so referring to them categorically as "lock and key" may eventually backfire.
4. P. 5, lines 105-109. Not clear what the distinction is between "reduced charging" and "mainly due to decreased overall yields of charged tRNA."
5. P. 9, lines 191-192. The dramatic destabilization of mitochondrial tRNAs might usefully appear

much earlier in the development, in particular in the introduction. The same holds for the fact that the mitochondrial alanyl-tRNA synthetase doesn't use the (G3:U70) (Page 8, line 175) base pair as an identity element. I found it troubling to be told these two, quite salient fact, so late in the paper.
6. P. 11, line 226. Is "for" supposed to be "from"?
7. P. 12, lines 250-254. These two sentences try to say too much, and I cannot exactly follow what the authors really want to say, despite the fact that it is closely related to their overall message. It is difficult to unpack the conflation of induced fit and negative determinants.

Charlie Carter, Professor of Biochemistry and Biophysics, UNC Chapel Hill

Reviewer #3 (Remarks to the Author):

Summary:

In the manuscript "Relaxed sequence constraints favor mutational freedom in idiosyncratic metazoan mitochondrial tRNAs", the authors formulate a model by which the extreme divergence of mitochondrial tRNAs in both sequence and structure, relative to their cytosolic counterparts. In their model, the deleterious effects of the inevitable accumulation of substitutions in mitochondrial tRNAs via Muller's ratchet are offset by the evolution of the nuclear-encoded mitochondrial synthetase to be more conducive to a broad array of mitochondrial tRNA sequences. The synthetases here maintain their specificity through recognition of structural elements of mitochondrial tRNA transcripts, rather than focusing on tRNA sequence features that cannot be expected to be conserved due to the lack of recombination of mitochondrial genomes. Overall, the authors do well to identify a system consistent with the divergence of mitochondrial tRNA genes, which is striking in comparison to cytosolic tRNA genes. Their model, as well as functional analyses to identify epistatic compensatory substitutions and their effects on tRNA charging, are well executed.

This manuscript introduces a unique and important example of cooperation between mitochondrial and nuclear gene sequences, and highlights the differences in modes of evolution of the two. It is a good fit for the journal and of general interest to readers.

Primary Concerns:

However, there are some concerns about potentially confounding factors that are not addressed:

1) The mitochondrial import of cytosolic tRNA transcripts is widespread across taxa (e.g. reviewed in Salinas-Giegé, et al, Int. J. Mol. Sci. 2015). This is particularly important if bilaterians import tRNAs at higher rates than their ancestors. This is consistent with some aspects of the model, including potentially decreased selective constraints on mitochondrial tRNAs to maintain their sequence integrity. However, this might also place decreased constraint on the mt-tRNA and the mt-AARS to be in sync with each other. Mitochondrial import should therefore be addressed in the manuscript.

2) Several mitochondrial tRNA genes do not exhibit the bizarre structural divergence highlighted here and instead are quite similar to cytosolic tRNA genes. There are some hypothetical mechanisms likely consistent with the authors' model that could be mentioned (differential mutation rates by locus, less stringent rules for recognition by certain AARSs). The focus throughout on tRNA-Ala does well as a clear example of the observed divergence, but addressing that many mt-tRNAs are exceptions to the model, or alternatively, predicted by the model due to other factors, would make this more impactful.

3) The manuscript points at an increased level of sequence divergence beginning at the Bilaterian divergence, but the figures make this look more like a peak at this point before trending back downwards. This pattern should be addressed. More generally, Figure 1 is pretty challenging to parse.

1b seems to show sequence divergence from the tips of the tree at the node connecting to other lineages. At a glance, one would assume these are reconstructed ancestral states, but they are not. Additionally, it is not clear how the estimates of nucleotide divergence and length changes map onto the tree at the bottom. I assume some lineages are not included, but it is not entirely clear. I recommend a significantly reworked figure and a much more detailed legend. One way this can be done is to have each lineage on the tree terminate at the present and then to display all relevant data for each lineage to the right of the branches.

4) The word "significantly" is used several times in your manuscript without reference to a statistical test or p-value. Most importantly, the point addressed by Figure 1 would be benefitted by e.g. a Wilcoxon Rank Sum test to demonstrate that mt-tRNAs show greater divergence following bilaterian expansion, or a Spearman Rank correlation to correlate divergence time and mt-tRNA sequence divergence, depending on the intended message. This is important to demonstrate because it is not completely clear based on the relatively greater abundance of data points after bilaterians that the stated trend is significant or that it continues through time.

5) The section "Mutational freedom associates with low complexity sequences" would benefit from reduction. It would be best if this section focused on a few of the most exemplary or interesting epistatic effects, and the rest could be moved to a supplemental section. The general readership of Nature Communications is unlikely to find each example sufficiently compelling to merit this extended treatment.

Minor Concerns:

1) In Figure 1, it would be best to assign a letter to each panel so as not to have to refer to portions as e.g. "the inset in the middle panel of Figure 1d". An explanation should also be provided on whether there is any significance for the branch lengths of e.g. Archaea, Bacteria, or whether only the divergence time (and not branch lengths) is meant to be indicated by this figure.

2) Figure 2 would benefit from being simplified. I would recommend only showing the WT, each of the six deletions, and the epistatic substitution(s) that restored each mutation genotype back to WT charging level. The rest would be better suited as a supplemental figure.

3) The paper concludes (lines 264-267) with a fairly specific order of events. The idea presented in the final paragraph of the manuscript is that mutational burden increased, and deleterious mutations in tRNAs selected for the evolution of aaRS recognition. However, it is also possible that the aaRS recognition evolved first and permitted the degradation of tRNA sequence via nearly-neutral divergence. The authors should consider being more circumspect in their explanation.

4) It would be beneficial to include some aspects of mitochondrial inheritance that differ from what is assumed under most cases of Muller's ratchet. For example, many cells contain multiple mitochondria, and there are quality control mechanisms including purifying selection to reduce the chances of transmitting harmful mtDNA to offspring (e.g. reviewed in Mishra and Chan, Nature Reviews, 2014). Differences in mitochondrial quality control across species may impact your model, and should be discussed.

Point-by-point response to Reviewer's comments

Reviewers' comments:

Reviewer #1 (Remarks to the Author):

The manuscript deals with the aspect of how mitochondrial translation (tRNA and aaRS) system adapts to the high mutational rate of the mitochondrial genome. Further, the authors have performed bioinformatic and biochemical studies based on which they claim that a G3:U70-independent recognition of tRNAAla is facilitated by key adaptations at the mitochondrial AlaRS level. Interestingly, they show that the aminoacylation barrier can be overcome by transplanting the variable arm (in particular the elbow region) thus identifying a mitochondrion specific tRNA identity element for tRNAAla system.
Overall, the manuscript is trying to link the above experimental observations to a larger phenomenon of mutational freedom in mt tRNAs which is possibly linked to the evolution of bilaterians. In my opinion, the claim is too tall and not sufficiently supported (see below). Also, the authors choose systems and examples arbitrarily to make their point while conveniently leaving out the exceptions!

Major concerns:
a) One of the major claims of the article is 'bilaterians mt tRNA have lost the identity determinants and are unstable'.

We do not explicitly state that "the identity determinants" were lost in bilaterian mt tRNAs. This would imply that all determinants are lost. To the contrary, we make clear that the anticodons, which often are identity determinants, have not been lost. To avoid any confusion on this point we have reworded the text on p. 3 of the revised manuscript.

• The unusual size and structure of mt tRNAs were previously noted (Suzuki et al, Annual Review of Genetics 2011, 45:1, 299-329; Salinas-Giege et al, Int. J. Mol. Sci. 2015, 15, 4518-4559).

Agree. But we are not sure why this is mentioned, as this was referenced in the introduction of our manuscript, as part of the background for our own analysis. In the revised manuscript we reiterate this point in the introduction and include additional references (p. 3 of the revised manuscript).

• Is there any rationale to analyse only 9 out of 20 amino acid-specific tRNAs? Supplementary Figure 1 includes tRNAs only from primates and mammals. What is the scenario with respect to lower bilaterians and non-bilaterians mt tRNAs?

We included the analyses for tRNAs of all amino-acid specificities in the revised manuscript. As anticipated, the additional examples further demonstrated the generality of our conclusions (shown in the revised Figure 1a and Supplementary Figure 1).

Further to the point, In the case of the original Supplementary Fig. 1 our purpose was to show the rapid evolutionary change that is still ongoing along the primary sequence of a particular case. We chose the well-studied mt $tRNA^{Ala}$ and compared it to the cytoplasmic counterparts. Here we showed that, despite the close relationships between various mammalian species, mammalian mitochondrial $tRNA^{Ala}$'s are divergent in sequence when compared to their conserved cytoplasmic counterparts.

We changed the legend of Supplementary Figure 1 (now Supplementary Fig. 2 in the revised manuscript) to make these points clearer. Moreover, we included sequence logos from additional examples, including vertebrates, echinoderms, various protostome lineages and non-bilaterians, which support the same

conclusion of exceptional divergence in bilaterian mt tRNAs. We also included a cloverleaf presentation of mt tRNA^{Ala} for the mammalian sequence alignment as a visual aid (Supplementary Fig. 2d)

• The loss of identity determinants in mt tRNAAla for AlaRS charging was reported in previous studies and an alternate mode of recognition was proposed. The G3:U70-independent mode of recognition by mt AlaRS was beautifully shown very recently by En-Duo Wang and co-workers, wherein multiple positions on the tRNA are recognized by the mitochondrial AlaRS (Zeng et al, Nucleic Acid Research 2019, 47:6, 3072-3085).

We very much appreciate the importance of the Zeng et al. work. However, to demonstrate G3:U70 independent tRNA recognition was not the focus of this manuscript. The vast majority of bilaterian lineages, including mammals, have lost the G:U wobble pair in the acceptor stem of mitochondrial tRNA^{Ala}'s. As elaborated in detail below (response to point d), our bioinformatics, mutational, and structural analysis is not focused on demonstrating loss of G:U recognition.
Rather, we were interested in the molecular mechanisms that replace G3:U70-specific recognition. In addition, we asked: what allows – or indeed favors – the unprecedented rate of sequence evolution and structural degeneration in bilaterian mitochondrial tRNAs. We added passages to further elaborate on this point and added additional references to the work by Zeng et al. (p. 3, 6, and 9 of the revised manuscript)

• The authors claim that G3:U70 is entirely lost from the metazoans mitochondria. In contradiction to this, it has been previously (Chihade et al, Biochemistry 1998, 37:25, 9193-9202) shown that C. elegans mitochondrial AlaRS uses G3:U70 as a determinant for charging the mt-tRNAAla. Like C. elegans, other bilaterians such as Albinaria caerulea and Ascaris suum also have a G3:U70 in the mt-tRNAAla. Hence the metazoan/bilaterian based argument does not hold good and unfortunately the authors neither choose to point these out or provide an explanation for these violations.

We should say that, at no point in the manuscript do we claim that G3:U70 is entirely lost from metazoan mitochondria. We stated, "the association with the G3:U70 identity element is lost almost entirely from mt-tRNA^{Ala} in bilaterian lineages".

Outside of bilaterian mitochondria, G3:U70 is virtually universally conserved in tRNA^{Ala} from bacteria to archaea and throughout eukaryotic lineages, including non-bilaterian mitochondria. However, a highly conserved G:U wobble pair in mt-tRNA^{Ala} is found in only 3 out of 24 bilaterian lineages (nematodes, nematomorpha, and xenacoelomorphs), corresponding to almost 90% loss in bilaterian lineages (in Hemichordates, G3:U70 is only partially conserved and in arthropods it is conserved as G2:U71 in Insects but not in chelicerata or crustacea). This indicates a selective regime within mitochondria which forces loss of G3:U70 in tRNA^{Ala} despite likewise ostensible pressure to retain it (Chihade et al, Biochemistry 1998, 37:25, 9193-9202 and Lovato et al, EMBO 2001, 20:4846-4853). Thus, exceptions such as those found in nematodes (including A. suum) and subgroups of arthropods emphasize the argument that strong evolutionary pressure favors the loss of G3:U70 in spite of strong tendencies to retain the pair.

We regret that our explanation for the diversity in bilaterian mt tRNA sequences was not made sufficiently clear. A central point of our argument, exemplified by the mammalian mitochondrial alanine system, is that mitochondrial recognition systems expand the functional sequence space for mt tRNAs to allow more sites to be mutationally neutral or near-neutral (constrained mostly by folding). Accordingly, G:U wobble pairs occur frequently as neutral or near-neutral transition states between A:U and G:C pairs throughout mt tRNA sequences, including the 3:70 position. Consistently, A. caerulea is the only

mollusk with G3:U70 in its mt $tRNA^{Ala}$, suggesting that it represents a neutral transitional state from the more common A3:U70. Indeed, G3:U70 is common in multiple other mollusk mt tRNA specificities such as mt $tRNA^{Arg}$ and mt $tRNA^{Ser}$. Similarly, mt $tRNA^{Arg}$ from the primate Hylobates lar (gibbon) and mt $tRNA^{Pro}$ from multiple other primate species contain G3:U70. These cases exemplify a central point of our argument: While in canonical systems $tRNA^{Ala}$ must contain G3:U70 and all other tRNAs must not contain G3:U70, in tRNAs of bilaterian mitochondrial recognition systems, the 3:70 position, like the other positions in the acceptor stem, becomes neutral to the presence or absence of G3:U70.

In the revised manuscript, we included a more extended discussion of the points raised above and made changes to include the examples mentioned by the reviewer (p. 5 and legend to Supplementary Figure 3 or the revised manuscript). Moreover, we included additional figures to better support our conclusions (Supplementary Figure 3).

• Previous work from the same group has shown that Drosophila melanogaster mtAlaRS recognizes the translocated G2:U71 of mt tRNAAla, while G3:U70 acts as a negative determinant for aminoacylation (Lovato et al, EMBO 2001, 20:4846-4853). Hence, the universality of G:U recognition in tRNAAla was put forward using the above data from the mitochondrial system as well. In light of these studies, the argument of bilaterian specific phenomenon becomes fragile and needs to be revisited.

Please see response above.

The systematic loss of G3:U70, or indeed any conserved G:U in the acceptor stem, occurs in the majority of lineages from all bilaterian phyla, including acoelomorphs. It does not occur in any other eukaryote lineage. In our opinion, this qualifies the loss of G3:U70 from mt $tRNA^{Ala}$ as a bilaterian-specific phenomenon.

b) The second major claim is the 'molecular adaptation of mtAlaRS to recognize the elbow region of the tRNA by creating a positive patch in the C-Ala domain, which is essential for aminoacylation'.
• The mutational analysis of the C-Ala domain (positive patch) shows that K822, R853, R860 are important for recognizing the tRNA. However, these residues are expected to be specific to mtAlaRS only, but (from supplementary figure 5) these residues are also conserved in ctAlaRSs. Thus, the claim for the appearance of the positive patch only in C-Ala domain of bilaterian mtAlaRS is questionable!
• R826 of mtAlaRS is not conserved but mutation to Alanine does affect the aminoacylation rate in one case. Does that mean that the proposed model is specific to only a very small subset of higher organisms?

We thank the reviewer for raising this issue. It is important to note several points:

1.) *With few exceptions, non-bilaterian cytoplasmic and mitochondrial AlaRSs are encoded by one nuclear gene. In contrast, they are encoded by two distinct genes in bilaterian lineages. Both the bilaterian ct AlaRS and mt AlaRS arose by gene duplication (they share a common non-bilaterian dual-localized AlaRS ancestor), presumably before the split of protostomes and deuterostomes. Thus, both genes are closely related and have a long history of joint evolution, likely reaching into early phases of bilaterian evolution, when it appears that mt tRNA structures started to diverge from the canonical state. Thus, it would not be surprising if they share certain traits involving recognition and stabilization of unstable mt tRNAs.*

2.) *Nowhere in the manuscript do we state that the appearance of particular positively charged residues is idiosyncratic to bilaterian mt AlaRS. Nor do we state that this particular positive patch in human mt AlaRS is universally conserved among bilaterian mt AlaRSs. Rather, while the tendency to diverge from the canonical mode of recognition is universal among protostome and deuterostome lineages, the solutions to adapt mt AlaRS to these changes likely occurred convergently, just as the G3:U70*

was likely lost from mt tRNA^{Ala} convergently in both lineages (see Supplementary Figure 3). The effect of mutations in human mitochondrial C-Ala on charging mt tRNA^{Ala} suggests an important contribution of these positively charged residues. In stark contrast, C-Ala is completely dispensable for charging tRNA^{Ala} by human ct AlaRS (see Sun, et al., PNAS 2016).

To make this point clearer, we included additional figures in the supplement (Supplementary Figure 7d-f) and reworded the corresponding passage (p. 8 of the revised text). Moreover, we emphasized the point that G3:U70 was likely lost convergently in various bilaterian lineages (p. 5 and Supplementary Figure 3 in the revised manuscript). Apart from our hypothesis that the common loss of G3:U70 in protostomes and deuterostomes, along with the loss of identity determinants in other mt tRNAs, high substitution rates and degeneration of structural determinants points toward a common selective pressure to accommodate mutational insults by altered aaRS/tRNA recognition systems, we do not speculate whether or not protostome mt aaRSs use similar or distinct molecular mechanisms to achieve this.

• The authors present the sequence alignment of AlaRS from different organisms. However, for mtAlaRS the list includes only chordates while leaving out the lower bilaterians/metazoans.

Non-bilaterian metazoans have only one gene for AlaRS. That gene encodes enzymes for both compartments. Thus, the sequences of S. cerevisiae and A. thaliana AlaRS represent both cytoplasmic and mitochondrial enzymes. We included additional sequences from several protostome species (revised Supplementary Figure 8).

• As the structure solved here is only the C-Ala domain and the interpretation is based on the full-length model stating "In particular, the mt AlaRS-specific Gly825 allows tight association between C-Ala and tRNA" may not be appropriate.

We thank the reviewer for pointing this out. We changed the passage accordingly to take these concerns into account (p. 9 in the revised text).

Do the authors mean that introducing G825 (along with M343, G444) in ctAlaRS is sufficient enough to charge mt-tRNAAla?

We do not assume that these changes would be sufficient to change specificity in ctAlaRS. Overall, human cytoplasmic and mitochondrial AlaRS have only 43% sequence identity (60% similarity) over their entire sequence lengths. Within the C-Ala domain this is further reduced to 28% identity (47% similarity). Among multiple other differences, vertebrate mt AlaRS contains an additional dipeptide in front of G444 and shows changes in the lengths of α-helices in the N-terminal portion of C-Ala. Moreover, comparison of the 3D structures of C-Ala from prokaryotic, human cytoplasmic and human mitochondrial AlaRS shows a significant reorientation of the C-terminal globular domain relative to the N-terminal helical domain in human cytoplasmic C-Ala. Specifically, human mt C-Ala is arranged like the bacterial and archaeal counterparts. On the one hand, this reorientation may explain the loss of a contribution to charging efficiency in ctAlaRS. On the other, it suggests that ct AlaRS would be unable to interact productively with mt-tRNA^{Ala}, even if G825, M343, and G444 would be introduced.

We included the structural comparison (overlay) between prokaryotic, human mt and ct C-Ala in Supplementary Figure 7d-f) and clarified this point in the accompanying figure legend.

c) The authors generalize the mechanism of "chaperon-like fitting" to the rest of the mt synthetases.
• A major premise of this manuscript is "We hypothesized that, in contrast to the rigid lock-key selection of cytoplasmic AARS-tRNA pairs, an adaptive chaperone-like fitting of the nuclear encoded synthetase with its mt tRNA partner made possible the mutational freedom of mt tRNAs". Due to the multidomain

architecture of aaRSs, they are shown to be involved in an induced-fit mode of enzyme-substrate interaction (Sherlin & Perona, Structure 2003, 11: 591–603). The induced fit/conformational selection of tRNA-synthetase recognition is so well known in the field and therefore it is difficult to imagine that the authors choose to present a totally different narrative!

We intended a different narrative on this point and should have been more mindful of the confusion it could cause. The concept of induced-fit was developed decades ago as a principle applied to many enzyme-substrate complexes, and generally referred to changes around the active site of the enzyme to accommodate the substrate. Along the same lines, Sherlin & Perona (Structure 2003, 11: 591–603) analyze how the induced-fit rearrangement of the aaRS's active site to ensure specificity in adenylate formation is coupled to binding of the canonical cognate tRNA. Here we have a large substrate, i.e. tRNA, and it is the substrate itself which undergoes a dramatic conformational change upon binding to the synthetase. A better analogy would be the synthetase as a chaperone, inducing folding of the tRNA.

We have restated this point in the revised text on several occasions (p. 11 and p. 13) and thank the reviewer for bringing this to our attention.

• In abstract the authors state "Outsourcing structural stability to nuclear-encoded aaRS partners" which is incorrect. By using the term "Outsourcing" does the authors mean the stability of mt aaRS is more compared to ct aaRS? Since the complex possibly is more stable, there is no 'outsourcing' happening here!

We thank the reviewer for this point. We regret that the terminology was confusing. By 'outsourcing' we meant that the tRNA itself did not have the elements needed to provide high efficiency of folding and stability. So, in these systems, another source had to be used as a 'helper'. We changed the abstract to meet the concerns by the reviewer.

• As correctly quoted (line 208), earlier studies have shown that mtSerRS uses tRNA elbow region for its charging. As the entire experimental proof for the current hypothesis is based on AlaRS and SerRS which do not follow the usual anticodon based tRNA recognition, any extrapolation or generalization to the rest of the mt synthetases may not be appropriate.

We thank the reviewer for raising this point that needs more clarification. We included additional passages in the revised text (p. 11 & p. 13).

As mentioned below, our interpretations were not meant to appear to be based on mt SerRS and mt AlaRS alone. Five points can be made:

1.) *A general trend across mt tRNAs for high rates of sequence evolution.*

2.) *A general trend to reduced thermal stability due to loss of canonical structural features of the elbow region. This is not only based on our own observations but has been reported for various mammalian mt tRNAs (e.g. Watanabe, Proc Jpn Acad Ser B Phys Biol Sci., 2010, 86(1): 11–39; Kumazawa, et al., J Biol Chem., 1989, 264(22):13005-11; Wang, et al., Biochem J, 2013, 453 (3): 455-465)*

3.) *A general trend in mt RNAs to lose otherwise highly conserved identity elements. Likewise, this is not limited to the alanine and serine systems, but was reported for the systems specific for leucine, aspartate and tyrosine (Sohm, et al., J Mol Biol 2003, 328: 995-1010; Sohm, et al, J Mol Biol 2004, 339: 17-29; Fender, et al, J Biol Chem 2006, 281: 15980-6; Fender, et al, Biochimie 2012, 94(5): 1090-97; Bonnefond, et al, RNA 2005, 11: 558-62).*

4.) *Analyses of other mitochondrial aaRS/tRNA systems such as mt LeuRS, mt AspRS, mt TyrRS (Sohm, et al., J Mol Biol 2003, 328: 995-1010; Sohm, et al, J Mol Biol 2004, 339: 17-29; Fender, et al, J Biol Chem 2006, 281: 15980-6; Fender, et al, Biochimie 2012, 94(5): 1090-97; Bonnefond, et al, RNA 2005, 11: 558-62) suggest a general ability of mt aaRSs to counteract the intrinsic instability of their mt tRNAs as a critical and unique feature of mitochondrial systems. For Example, human mt AspRS was shown to charge mt tRNA^{Asp}, despite the absence of D-loop-T-loop interactions, while E. coli AspRS is virtually inactive on the same substrate (Fender, et al, J Biol Chem 2006, 281: 15980-6; Fender, et al, Biochimie 2012, 94(5): 1090-97). This is consistent with the uniqueness of structural instability among bilaterian mt-tRNAs.*

5.) A unilateral aminoacylation barrier between mitochondrial and bacterial (canonical) recognition system that allows mammalian mt aaRSs to aminoacylate canonical tRNAs, while canonical aaRSs *are generally unable to aminoacylate even cognate mt tRNAs (Fender, et al, Biochimie 2012, 94(5): 1090-97; Kumazawa, et al., J Biochem 1991, 109:421-7).*

We have modified the text to add more clarity on this point (p. 11, 12, and 13).

• Earlier studies have shown that in case of mitochondria the number of determinants for aminoacylation are reduced but not rewired fully (Suzuki et al, Annual Review of Genetics 2011, 45:1, 299-329).

We agree.

As mentioned above, we do not state that all identity elements have been lost or rewired in bilaterian mitochondrial tRNAs. It is obvious that any synthetase that uses the anticodon of its tRNA substrate as an identity determinant is likely to continue to do so in bilaterian mitochondria. The anticodon, due to its role in ribosomal mRNA decoding, yields a significantly stronger purifying selection than the rest of the tRNA body. We also did not exclude the possibility that, in some cases, mitochondrial aaRS/tRNA pairs retained individual nucleotides, e.g. the discriminator base that is not involved in base-pairing interactions, as identity elements in addition to the anticodon loop.

We focused on mt AlaRS and mt SerRS explicitly because they use anticodon-independent recognition. The rationale is that, because they do not use the anticodon for recognition, they cannot, like other aaRSs do, rely on the selective constraint by the codon-anticodon interaction in mRNA decoding to preserve their identity elements. Instead, in the canonical recognition system AlaRS and SerRS use unique sequence and structural features that are embedded into the structural framework of their tRNA substrates that is universal among canonical tRNAs. Since they cannot fall back on the uniqueness of their tRNA's anticodons, which remain (almost) invariant regardless of how strong the mutation pressure is, the absence of sequence specific recognition in these two systems is of particular interest. To put it differently: If E. coli AspRS stops recognizing G73 and G2:C71 (as observed for mt AspRS), it can in principle still use anticodon recognition to maintain a minimum degree of selectivity between cognate and non-cognate tRNAs. If E. coli AlaRS stops recognizing G3:U70 and instead accepts G3:C70, the entire canonical recognition system would need to be changed, as 19 isoacceptors, with anticodons for 10 different amino acids, have G3:C70 embedded into the same canonical tRNA fold that also defines E. coli tRNA^{Ala} and would become substrates for AlaRS. This exact situation, i.e. that AlaRS has stopped recognizing G3:U70, is present in the human mt AlaRS/tRNA^{Ala} system (and the majority of bilaterian lineages), and our results indicate that, at least in the human system, there is no alternative base-pair or set of base-pairs in the acceptor stem that take over the original role of G3:U70. The same general considerations apply to SerRS. The interdependent nature of aminoacylation systems implies that changing one (or several) subsystems necessarily requires adjustments in all other subsystems. This is particularly true for changes in anticodon-independent aaRSs.

However, as stated above, the generalization of our model to other mitochondrial aaRS/tRNA pairs is not based exclusively on the analysis of AlaRS and SerRS. First, the sequence analysis of mt tRNAs indicates a general increase of sequence divergence, loss of sequence identity elements, and deterioration of the canonical elbow region. Thus, the necessity for nuclear-encoded mt aaRSs to adapt to the changed reality in their mt-tRNA substrates is general among mitochondrial aminoacylation systems.

Moreover, as we discuss on p.10 of the original manuscript (p. 11 in the revised text), several other mammalian mitochondrial aaRSs have an increased flexibility towards the instability of their mt-tRNA substrates. These include human mtLeuRS and mtAspRS (Sohm, et al., J Mol Biol 2003, 328: 995-1010; Sohm, et al, J Mol Biol 2004, 339: 17-29; Fender, et al, J Biol Chem 2006, 281: 15980-6; Fender, et al, Biochimie 2012, 94(5): 1090-97).

We revised the text to more clearly state these points (p. 11 and p. 13).

d) tRNA sequence-independent recognition of mt tRNAAla by mt AlaRS.

• In line 137, the authors contradict the recent finding (Zeng et al, Nucleic Acid Research 2019, 47:6) and state that the tRNA recognition by mtAlaRS is based on the structure but not the sequence of the mt tRNAAla. Given the inadequate structural and biochemical data available, it is premature to say that tRNA structure plays an important role but not sequence. Perhaps, it appears that it is the combination of both which is recognized by mtAlaRS. In any case, the current work does not go beyond what has been published by Zeng et al. NAR 2019 in this aspect.

We highly respect and acknowledge the Zeng et al. (NAR 2019, 47:6) study from the En-Duo Wang's laboratory. Both our work and theirs analyze the same model system. We see no conflict in the data sets. However, this work expanded substantially on the excellent prior work and, in part for that reason, led to very different conclusions.

According to the reviewer, our work does not go beyond the work by Zeng et al. (NAR 2019). First, it should be noted, as discussed above, that the focus of our study is very different from that in Zeng et al. We address not only the question of whether G3:U70 is lost from human mt tRNA^{Ala} as a recognition element, but also these questions: 1) How its loss was compensated in human mitochondrial aminoacylation systems; 2) How this adjustment in mitochondrial recognition systems is related to high evolutionary rates in mtDNA; 3) Why was G3:U70 (together with other identity elements) lost only in one particular group of eukaryotes, namely bilaterians?

Zeng et al. address only the first question, stating that "hmtAlaRS recognizes tRNAAla in a G3-U70-independent manner, with several nucleotides in the acceptor stem being critical elements" (Abstract on p. 3072 and similarly stated in the title). Apart from the conclusion that the G3:U70 identity element is not used by human mtAlaRS (which is expected from sequence analysis alone, given the absence of a G3:U70 base-pair in human mt-tRNA^{Ala} and the absence of a conserved G:U base-pair in the acceptor stem of known mt tRNA^{Ala} sequences within the entire primate lineage), the central conclusion by Zeng et al. is that "Three base pairs (A1-U72, A2-U71 and G4-C69) are likely to be the most important base pairs in the acceptor stem for aminoacylation, while A5-U68 was necessary but does not function as a recognition determinant" (p. 3078 in Zeng et al.). In other words, base pairs A1:U72, A2:U71 and G4:C69 are considered by Zeng et al. as recognition determinants in human mt-tRNA^{Ala} that replaced G3:U70.

In the light of our additional results and analysis, we have come to different conclusions. More specifically:

1.) *The G1:C72 mutation indeed shows a marked reduction in charging activity (which we also observe). However, the U1:A72 mutation shows an only 3-fold reduction in k_{cat} and only 2-fold lower catalytic*

efficiency than wild-type tRNA, suggesting that A1:U72 is not required for direct, sequence-specific recognition. Consistent with the data for U1:A72 (but not the interpretation by Zeng et al.), we find that the G1:C72 mutation can be compensated by distant second site mutations. The effect of G1:C72 in the context of an otherwise wild type tRNA is thus indirect and A1:U72 not a critical direct recognition element akin to G3:U70. Consistent with this analysis, A1:U72 is not conserved among all mammalian species and can be changed in different sequence contexts, e.g. to G1:U72 in Pongo abelii (Orangutan) or to G1:C72 (together with A4:U68) in Tachyglossus aculeatus (Echidna).

2.) *In Zeng et al. the A2:U71 to U2:A71 mutation drastically reduced plateau levels of charged tRNA. Yet, no effect was observed for mutation of A2:U71 to G2:C71 (k_{cat}/K_M of 1.17 vs 1.12 $mM^{-1}min^{-1}$). This is consistent with our data for mutations in this base-pair, and, in our interpretation, argues against direct sequence-specific recognition of A2:U71. The effect of the transversion mutation U2:A71 is thus most likely rather due to the inversion of the purine:pyrimidine geometry, which disrupts base stacking and thus stability within the two acceptor stem strands (the R:Y geometry in the first five base-pairs of the acceptor stem is indeed absolutely conserved in mammalian mt $tRNA^{Ala}$ sequences), and not due to the loss of specific atomic determinants. Consistently, the 2:71 base pair exists in mammals either as A2:U71 (e.g. human), G2:C71 (e.g. Hippopotamus amphibious and Chalinolobus tuberculatus), or G2:U71 (e.g. the primate Chlorocebus aethiops).*

3.) *The effect of G4:C69 mutation was again shown by the transversion mutation U4:A69. Again, this violates the absolutely conserved R:Y geometry in mammalian mt $tRNA^{Ala}$ and is likely to alter the stability/geometry of the acceptor stem. We find that mutation to G4:U69, which retains R:Y symmetry but alters the Watson Crick base pair to a wobble pair, is compatible with charging by AlaRS, if the structural instability it causes is compensated by distant second site mutations. Both G4:U69 and A4:U69 occur in mammalian mt $tRNA^{Ala}$'s within altered overall sequence contexts (e.g. Dasyurus hallucatus, Tachyglossus aculeatus, Caenolestes fuliginosus).*

4.) *None of the elements identified by Zeng et al. is unique to human mt $tRNA^{Ala}$; all are present in multiple other species of human mt tRNAs. In particular, human mt $tRNA^{Asp}$ is identical to human mt $tRNA^{Ala}$ in the discriminator base and the first three base-pairs of the acceptor stem, an observation that motivated our experiments with that tRNA. Additionally, in human mt $tRNA^{Asp}$ base-pair 4:68 can be present either as G4:U68 or G4:C68 due to polymorphisms in position 68. The human population thus contains mt $tRNA^{Asp}$ which is identical to mt $tRNA^{Ala}$ in all positions considered as identity elements by Zeng et al. As we describe in more detail above (response to point c), loss of G3:U70 recognition by AlaRS necessarily poses the critical question how misaminoacylation of non-cognate tRNAs is prevented. How human mt AlaRS discriminates against mt $tRNA^{Asp}$ and other mt tRNAs with similar acceptor stem is not explained by the model proposed by Zeng et al.*

In summary, the interpretation by Zeng et al. of their data does not provide an explanation for how mt tRNAs with overlapping sequence identities are discriminated between and does not provide a rationale for why an otherwise absolutely conserved identity element should be replaced by several other only partially conserved elements. Their data are however consistent with our interpretation that mt AlaRS does not rely on specific atomic determinants in the acceptor stem or in other parts of the tRNA, but instead on the overall geometry and folding ability of its tRNA substrate. We have extended the data from Zeng et al. by showing that every base pair in the acceptor stem can be altered without losing charging activity, if the sequence context allows correct folding of the tRNA. Our data show that the idiosyncratic elbow region and its instability are critical for recognition of mt $tRNA^{Ala}$ through the C-Ala domain of mtAlaRS as well as for the discrimination of non-cognate tRNAs. Most importantly, though, our model provides a rationale for the unique loss of G3:U70 throughout bilaterian lineages and the ability of their mt aaRS/tRNA systems to withstand the high mutation pressure that was associated with bilaterian

evolution. Our model thus provides a consistent framework for the interpretation of previously published data on mammalian mt aaRS/tRNA recognition systems, the reduced structural stability in mt tRNAs, the degeneration of the canonical elbow region, and the frequent loss of canonical identity elements.

In our opinion, much more important than the question of whether G3:U70 is lost from mt-tRNA^{Ala} as a recognition element are the questions how this loss was compensated in mitochondrial aminoacylation systems and why it occurs in specifically in one particular group of eukaryotes, namely bilaterians?

We have included additional references to Zeng et al. in the revised manuscript (p. 3 and 6).

Other concerns:
• Line 23: ‘aminoacyl tRNA’ should be written as ‘aminoacyl-tRNA’ in the abstract.

We thank the reviewer for pointing this out. We corrected the mistake.

• Line 26: ‘Or’ needs to be replaced by ‘Our’ in the abstract.

We changed ‘Or’ to ‘Our’.

• Line 136: The non-reverting mutation of mt tRNAAla U54/58 is not shown in Figure 3c or table1-3.

A54/A58 is the mutation that recovers charging activity without reverting back to the original sequence. Actually, this mutation was in the original manuscript (Figure 3c and Table 2; Figure 2c and Table 1 in the revised manuscript).

• In Figure 5c the activity is plotted relative to tRNAAsp, instead, these values need to be compared with mt tRNAAla.

We agree with the reviewer that we should include the comparison to mt tRNA^{Ala}. We therefore included the comparison of relative activities both in Figure 5 and Table 3.

• Line 210: The authors do not include Ser in the earlier bioinformatics analysis (Line 70), but use them as a proof of concept.
• Here the authors use SerRS as a proof of concept. Is it possible to show with other more canonical mt synthetases?

We included bioinformatics analysis of tRNA^{Ser} in the revised manuscript (see Figure 1a and Supplementary Figure 1).

If “more canonical” means the use of the anticodon as identity determinant by the mt aaRSs, then we refer to the example of mt AspRS cited in the manuscript (Fender, et al, J Biol Chem 2006, 281: 15980-6; Fender, et al, Biochimie 2012, 94(5): 1090-97). However, AlaRS and SerRS are particularly interesting and important for the presented concept, due to their lack of anticodon recognition (please see also response to point c) raised by the reviewer).

• Line 502: The reference volume and page numbers are incorrect.

We apologize for the inaccuracy in the citation. This citation was included when the paper was published online and thus before volume and page numbers were assigned. The error was corrected.

• Table 2: the tRNA mutation ‘G1:C73/C62/C68’ is incorrect, and should be changed to

‘G1:C72/C62/C68’

Again, we thank the reviewer for pointing this out to us. The error was corrected.

Reviewer #2 (Remarks to the Author):

This important manuscript describes new phenomena relevant to understanding how the emergence of mitochondria as an efficient energy source impacted the evolutionary emergence of higher-order animals with bilateral symmetry. The notional steps in the argument are these: (i) mitochondria reproduce asexually and without recombination, making them potentially vulnerable to irreversible accumulation of deleterious mutations (i.e. Muller’s ratchet); (ii) mitochondrial transfer RNAs exhibit extraordinary variations in size and sequence, compared to the more stable cytoplasmic tRNAs; (iii) mitochondrial tRNA variants are both structurally less complex and relatively unstable; (iv) the cognate mitochondrial aminoacyl-tRNA synthetase structures—encoded in the nucleus—compensate these vulnerabilities by developing unusual induced-fit binding mechanisms quite distinct from those of the cytoplasmic cognate pairs. As each of these statements opens substantial questions, the manuscript is necessarily highly speculative. Nonetheless, the authors do a credible job shoring up each conclusion.

The manuscript combines several diverse threads from the Schimmel laboratory, including their interest in disease-causing mutations mitochondrial tRNAs, significant markers of the major branch points in the evolution of higher organisms, and their long-standing contributions to the structural enzymology of aminoacyl-tRNA synthetases. The paper should be highly interesting to a broad spectrum of readers.

The work described was motivated by detailed studies of the disease-causing mutations in the mitochondrial tRNAAla. The salient conclusion from an impressive number of observations and related experiments in this manuscript is that these disease-causing mutations can be compensated by mutations at other sites (epistasis) as well as by transformed binding interfaces with the cognate synthetase. In particular, chimeric tRNAAla molecules in which regions of the cytoplasmic tRNAAla are replaced by their counterparts from mitochondrial tRNAAla restored the ability to acylate the cytoplasmic chimeras. Because these experiments suggested that context, rather than sequence determined binding, an induced-fit binding mechanism involving the elbow region appears critical for productive binding.

The authors describe structural data pertinent to this phenomenon by solving the domain responsible for binding the elbow from the mitochondrial synthetase, and combine it with artful homology modeling to show the extensive co-adaptation that shifted the recognition mechanism from a highly focused and sequence-dependent recognition of the (G3:U70) base pair, used almost universally by AlaRSs, to a more sequence-independent recognition of the tRNA elbow in the human mitochondrial AlaRS•tRNAAla complex.

Similar, but more limited analysis of mitochondrial tRNASer reveals comparable changes, suggesting that the mitochondrial alanine system is representative of a detailed distinction between how tRNAs are recognized by cytoplasmic aminoacyl-tRNA synthetases and (nuclear encoded) mitochondrial synthetases. An imaginative speculation by the authors is that the nuclear synthetase genes for mitochondrial synthetases co-adapt to the rapid sequence evolution of mitochondrial tRNA synthetase genes by developing chaperone-like induced-fit recognition mechanisms that preserve the coding specificity necessary to ensure survival of the bilateran energy-generating mitochondria, whose genomic DNA must evolve under higher oxygen tension.

The paper is quite well-written and conclusions are fully documented. The authors might consider the following suggestions:

1. P. 2, line 26. “Or” should probably be “Our”.

Thank you for pointing this out. We changed ‘Or’ to ‘Our’.

2. P. 2, line 27. “evolution of animal complexity”. Are there important differences between the bilateran mt tRNA sequences and those from the plastids of other higher organisms (i.e. chloroplasts).

This is indeed a very interesting question. In comparison to mitochondria the information on fully annotated plastid (cp) tRNAs is limited and the relatively small number of studies on this topic do not allow a comprehensive answer to the question. In general, plastid genomes of land plants encode a larger number of tRNA genes (usually 30-40 genes for 25-30 isoaccepting families) than bilaterian mtDNA (22). Despite the larger number of genes, many plastid genomes were found to lack tRNAs of individual specificities, suggesting their import from the cytosol (which has not been demonstrated so far) (Michaud, et al., The Plant Journal, 2011, 66: 80-93). Overall, the evolutionary history of cp tRNAs appears to be more diverse, in some cases with cpDNA encoded tRNAs derived from the plant mtDNA genome. Although unusual cp tRNAs do seem to exist (e.g. Mohanta, et al., BMC Plant Biol., 2019, 19: 39), the structures of plant cp tRNAs are generally very closely related to their bacterial counterparts, with low A/U content, stable base-pairing in stem regions, average lengths of 76 nt in monocots (angiosperms), and with structural determinants in the D- and T-loop and identity elements highly conserved. For example, the G3:U70 element and the long variable arm are conserved in cp tRNA^{Ala} and cp tRNA^{Ser} species, respectively, from Green Algae and Dinoflagellates to Angiosperms (see below). Thus, despite the relatively limited information available, a general loss of canonical characteristics in plant cp tRNAs, akin to bilaterian mt tRNAs, seems unlikely. This is consistent with the conservation of canonical features in plant mt tRNAs that we observe. It is important to note that plant chloroplast genomes do not show elevated mutation rates compared to the nuclear genome. Instead, many plant cpDNA genomes appear to have lower mutation rates than the nuclear and mitochondrial genomes (e.g. Wolfe, et al., PNAS, 1987, 84(24):9054-8; Plamer, et al., J Mol Evol., 1988, 28(1-2):87-97). Thus, plant cp tRNA recognition systems are not exposed to the same intense mutational pressure under which bilaterian mt tRNA systems evolved.

As we would like to keep the manuscript focused on the comparison between mitochondrial and cytoplasmic tRNAs, we would prefer to not include a discussion of plant cp tRNAs. We hope that this acceptable to the reviewer. To exemplify the close adherence of cp tRNA to canonical tRNA features and their difference to bilaterian mt tRNAs, we included a short alignment of cp tRNA^{Ala}'s (green) with the nuclear-encoded counterpart from A. thaliana (blue), the cyanobacterium T. elongates (black) and mtDNA encoded mt tRNA^{Ala} (orange) from human and octopus (G3:U70 are highlighted in red, G18, G19 and the TψC trinucleotide are highlighted in bold black).

```
cptRNA-Ala_Helicosporidium_sp        GGGAATGTAGTTTCAGTTGGTAGAACGTTGCTTTTGCAAAGCAATAGACAGCGGTTCAAGTCCGCTCATTTCCA
cptRNA-Ala_Karlodinium_veneficum     GGGAGTATAGCT-CAGATGGTAGAGCACTGCTTTTGCACAGCAGATGACATCGGTTCGAATCCGTTTACTTCCA
cptRNA-Ala_Daucus_setulosus          GGGGATATAGCT-CAGTTGGTAGAGCTCCGCTCTTGCAAGGCGGATGTCAGCGGTTCGAGTCCGCTTATCTCCA
cptRNA-Ala_Arabidopsis_thaliana      GGGGATATAGCT-CAGTTGGTAGAGCTCCGCTCTTGCAAGGCGGATGTCAGCGGTTCGAGTCCGCTTATCTCCA
cptRNA-Ala_Oryza_sativa              GGGGATATAGCT-CAGTTGGTAGAGCTCCGCTCTTGCAAGGCGGATGTCAGCGGTTCGAGTCCGCTTATCTCCA
cptRNA-Ala_Quercus_rubra             GGGGATATAGCT-CAGTTGGTAGAGCTCCGCTCTTGCAAGGCGGATGTCAGCGGTTCGAGTCCGCTTATCTCCA
cytRNA-Ala_T.elongatus               GGGGGTATAGCT-CAGTTGGTAGAGCACCTGCTTTGCACGCAGGGGGTCAGCGGTTCGAGTCCGCTTACCTCCA
nctRNA-Ala_Arabidopsis_thaliana      GGGGATGTAGCT-CATATGGTAGAGCGCTCGCTTTGCATGCGAGAGGCACAGGGTTCGATTCCCTGCATCTCCA

mttRNA-Ala_H.sapiens                 AAGGGCTTAGCT-TAA--TT-AAAGTGGCTGATTTGCGTTCAGTTGA-TGCAGAGTGGGGTTTTGCAGTCCTTA
mttRNA-Ala_O.vulgaris                GGTATTATATTT-TAT--ATAAGAAAATTTAACTTGCAATTAAAAAG-TAATAACT--AATTTATTTAATACCA
```

3. P. 3, lines 59-62. The authors run the risk of making to sharp a distinction here between “lock and key” and induced-fit. Most of the cytoplasmic synthetases are likely to exhibit induced fit in various ways as well, so referring to them categorically as “lock and key” may eventually backfire.

We understand the concern and changed the corresponding passage on p. 3 accordingly and included a brief discussion on this point on p. 13 of the revised text.

4. P. 5, lines 105-109. Not clear what the distinction is between "reduced charging" and "mainly due to decreased overall yields of charged tRNA."

Thank you for pointing this out as a potential source of confusion. We meant was that reduced charging of the mutant tRNAs could either be due to the loss of a direct recognition element by the mutation or due to misfolding because of destabilization of the wild type fold. Since the mutations reduced the final yield of charged tRNA and the compensatory second-site mutations restored original yields, we concluded that the initial mutations had not disrupted a critical direct aaRS interaction but had instead increased the relative abundance of misfolded tRNAs. We rewrote the corresponding passage in the revised manuscript to avoid confusion (p. 7 in the revised text).

5. P. 9, lines 191-192. The dramatic destabilization of mitochondrial tRNAs might usefully appear much earlier in the development, in particular in the introduction. The same holds for the fact that the mitochondrial alanyl-tRNA synthetase doesn't use the (G3:U70) (Page 8, line 175) base pair as an identity element. I found it troubling to be told these two, quite salient fact, so late in the paper.

We agree that both aspects, dramatic destabilization and loss of G3:U70 as identity element are important and should be mentioned earlier and more prominently in the manuscript. We included both aspects more prominently in the results section (p. 5) and the introduction (p. 3) as suggested.

6. P. 11, line 226. Is "for" supposed to be "from"?

Yes, it was meant to read "from". Thank you for pointing this out. The mistake was corrected.

7. P. 12, lines 250-254. These two sentences try to say too much, and I cannot exactly follow what the authors really want to say, despite the fact that it is closely related to their overall message. It is difficult to unpack the conflation of induced fit and negative determinants.

Thank you for bringing this up. We rewrote both sentences in a way that we hope makes it easier to follow what we intend to say (p. 12-13).

Charlie Carter, Professor of Biochemistry and Biophysics, UNC Chapel Hill

Reviewer #3 (Remarks to the Author):

Summary:

In the manuscript "Relaxed sequence constraints favor mutational freedom in idiosyncratic metazoan mitochondrial tRNAs", the authors formulate a model by which the extreme divergence of mitochondrial tRNAs in both sequence and structure, relative to their cytosolic counterparts. In their model, the deleterious effects of the inevitable accumulation of substitutions in mitochondrial tRNAs via Muller's ratchet are offset by the evolution of the nuclear-encoded mitochondrial synthetase to be more conducive to a broad array of mitochondrial tRNA sequences. The synthetases here maintain their specificity through recognition of structural elements of mitochondrial tRNA transcripts, rather than focusing on tRNA sequence features that cannot be expected to be conserved due to the lack of recombination of

mitochondrial genomes. Overall, the authors do well to identify a system consistent with the divergence of mitochondrial tRNA genes, which is striking in comparison to cytosolic tRNA genes. Their model, as well as functional analyses to identify epistatic compensatory substitutions and their effects on tRNA charging, are well executed.

This manuscript introduces a unique and important example of cooperation between mitochondrial and nuclear gene sequences, and highlights the differences in modes of evolution of the two. It is a good fit for the journal and of general interest to readers.

Primary Concerns:

However, there are some concerns about potentially confounding factors that are not addressed:

1) The mitochondrial import of cytosolic tRNA transcripts is widespread across taxa (e.g. reviewed in Salinas-Giegé, et al, Int. J. Mol. Sci. 2015). This is particularly important if bilaterians import tRNAs at higher rates than their ancestors. This is consistent with some aspects of the model, including potentially decreased selective constraints on mitochondrial tRNAs to maintain their sequence integrity. However, this might also place decreased constraint on the mt-tRNA and the mt-AARS to be in sync with each other. Mitochondrial import should therefore be addressed in the manuscript.

This is indeed an important point. We did not include a discussion of mitochondrial tRNA import in this manuscript because of the limited information available for bilaterian species. Similar to other cytosolic RNAs for which import into mammalian mitochondria was reported, such as 5S rRNA and H1 RNA, the in vivo abundance and physiological relevance of cytosolic tRNAs in bilaterian mitochondria remains unknown and controversial. Against this background of limited information and contrasting views in the field on the matter, we did not feel confident to include a comprehensive discussion of mitochondrial tRNA import in bilaterians.

We included a paragraph on cytoplasmic tRNA import in the discussion section of the revised manuscript (p. 13). If the reviewer considers it necessary to extend this section, we of course could further elaborate on this point.

2) Several mitochondrial tRNA genes do not exhibit the bizarre structural divergence highlighted here and instead are quite similar to cytosolic tRNA genes. There are some hypothetical mechanisms likely consistent with the authors' model that could be mentioned (differential mutation rates by locus, less stringent rules for recognition by certain AARSs). The focus throughout on tRNA-Ala does well as a clear example of the observed divergence, but addressing that many mt-tRNAs are exceptions to the model, or alternatively, predicted by the model due to other factors, would make this more impactful.

Thank you for bringing this up. We believe that, at least in some cases, additional selective pressures also come into play. A case in point is the more canonical appearing mt $tRNA^{Gln}$ in vertebrates. Interestingly, there is no dedicated mitochondrial glutaminyl-tRNA synthetase (GlnRS). In this instance, mt $tRNA^{Gln}$ is first charged by mitochondrial glutamyl-tRNA synthetase (GluRS) and the attached glutamyl-group is then modified by the mitochondrial GatCAB complex to give a glutaminyl-group. Thus, both mt GluRS and the GatCAB complex have to recognize mt $tRNA^{Gln}$. This constraint may be strong enough to force mt $tRNA^{Gln}$ to remain more canonical in its structure. Constraints through interactions with multiple other protein partners may thus be a common feature among more canonical mt tRNA species. Moreover, another factor may indeed be the location of mt tRNA genes on the mtDNA genome, due to differences in transcription and processing of primary transcripts. We did observe a statistically significant difference between vertebrate mt tRNAs encoded by the light (L) strand ("heavy" mt tRNAs) vs heavy (H) strand-encoded ("light") mt tRNAs (see figure below). A reason for the observed stronger conservation of

"heavy" mt tRNAs could be the asymmetrical processing mechanisms of H- and L-stands, where defects due to mutations in L-strand (heavy) mt tRNAs can reduce levels of all other L-strand tRNAs, while processing defects in a mutated H-strand tRNA do not have the same global effects (Zhao, et al. Nucleic Acids Res 2019, 47:10340-10356). This would put more selective pressure on heavy mt tRNAs. Similar effects on processing and/or posttranscriptional processing may apply to a few other mitochondrial tRNAs, where a more conventional structure is retained.

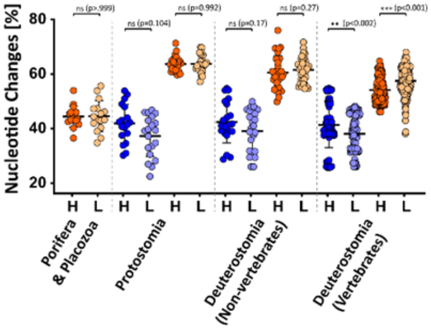

Comparison of nucleotide changes in mt tRNAs encoded by the heavy and light mtDNA strands. The light mtDNA strand encodes "heavy" (H) mt tRNAs (dark colors), while the heavy strand encodes "light" (L) mt tRNAs (light colors). The distinction into H and L tRNAs is only meaningful for mt tRNAs (orange), while the distinction is not meaningful for ct tRNAs (blue), which are grouped into H and L according to their mitochondrial counterparts. While Non-bilaterian, protostome, and non-vertebrate deuterostome mt tRNAs do not show differences between the two groups, vertebrates show significantly lower sequence change in heavy mt tRNAs (specific for Ala, Asn, Cys, Gln, Glu, Pro, Ser(UGA), Tyr) than in light mt tRNAs ($p<0.001$).

It is important to note that the more canonical mt tRNAs are only found in deuterostomes, whereas protostomes show strong degeneration throughout all mt tRNA species. The constraints acting on some vertebrate mt tRNAs thus do not necessarily apply also to protostome mt tRNAs, or at least not to the same degree. Moreover, even the 'more canonical' vertebrate mt tRNAs show the same trend toward increased A/U content and overall sequence divergence from their canonical counterparts as observed for other, less canonical mt tRNAs and altered geometries between structural domains. For example, mt tRNA^{Leu2} is one of the more canonical mt tRNAs in vertebrates in terms of secondary structure. Yet, mt tRNA^{Leu2} is highly divergent in terms of sequence and is only partially folded in solution, requiring mt LeuRS for stable folding (Sohm, et al., J Mol Biol 2004, 339:17-29). Moreover, while mt tRNA^{Leu2} has lost the otherwise highly conserved long variable arm found in all canonical tRNA^{Leu} variants, it is the only mt tRNAs with a dramatically larger D-loop (9-13 nucleotides), which may serve as structural anti-determinant for some non-cognate mt aaRSs. Similarly, mt tRNA^{Ser2} exhibits canonical-like elements in the D- and T-loops and yet shows topological idiosyncrasies in form of shortened linkers between domains or an extended anticodon-stem.

We initially included a discussion of the above points in the revised manuscript. Yet we ultimately felt that the details that a comprehensive discussion of these subjects requires goes too far beyond the focus of this work and may distract from what we consider the major point, i.e. that bilaterian mt tRNAs, but not non-bilaterian mt tRNAs, on average show a drastically increased tendency for the degeneration of canonical structure elements, thermodynamic instability, and loss of identity elements. We hope that the reviewer understands our concerns and our decision not to include this discussion. Should the reviewer still think that such a discussion should be included, we would be happy to include it as a detailed supplementary section.

3) The manuscript points at an increased level of sequence divergence beginning at the Bilaterian divergence, but the figures make this look more like a peak at this point before trending back downwards. This pattern should be addressed. More generally, Figure 1 is pretty challenging to parse. 1b seems to show sequence divergence from the tips of the tree at the node connecting to other lineages. At a glance, one would assume these are reconstructed ancestral states, but they are not. Additionally, it is not clear how the estimates of nucleotide divergence and length changes map onto the tree at the bottom. I assume some lineages are not included, but it is not entirely clear. I recommend a significantly reworked figure

and a much more detailed legend. One way this can be done is to have each lineage on the tree terminate at the present and then to display all relevant data for each lineage to the right of the branches.

Thank you for this valuable suggestion. We followed the reviewer's instructions to rearrange Figure 1 and include a more detailed legend. In particular, we addressed the observed trend mentioned by the reviewer for Fig. 1b and redrew the tree at the bottom. Also, we introduced an alternative dot plot of the data in Supplementary Figure 1 to apply statistical tests (see below).

4) The word "significantly" is used several times in your manuscript without reference to a statistical test or p-value. Most importantly, the point addressed by Figure 1 would be benefitted by e.g. a Wilcoxon Rank Sum test to demonstrate that mt-tRNAs show greater divergence following bilaterian expansion, or a Spearman Rank correlation to correlate divergence time and mt-tRNA sequence divergence, depending on the intended message. This is important to demonstrate because it is not completely clear based on the relatively greater abundance of data points after bilaterians that the stated trend is significant or that it continues through time.

Thank you for bringing this up. We included statistical tests and p-values as suggested by the reviewer (Supplementary Figure 1).

5) The section "Mutational freedom associates with low complexity sequences" would benefit from reduction. It would be best if this section focused on a few of the most exemplary or interesting epistatic effects, and the rest could be moved to a supplemental section. The general readership of Nature Communications is unlikely to find each example sufficiently compelling to merit this extended treatment.

Thank you for this suggestion. We rewrote the passage for the revised version of the manuscript as suggested.

Minor Concerns:

1) In Figure 1, it would be best to assign a letter to each panel so as not to have to refer to portions as e.g. "the inset in the middle panel of Figure 1d". An explanation should also be provided on whether there is any significance for the branch lengths of e.g. Archaea, Bacteria, or whether only the divergence time (and not branch lengths) is meant to be indicated by this figure.

Thank you for pointing this out. We changed the figure accordingly. Indeed, the tree was only meant as a visual guide to the divergence time, without particular significance for the branch length. We adjusted the figure and legend accordingly to make this point clearer. Some of the data/panels were moved to Supplementary Figure 2 for more clarity in the main figure.

2) Figure 2 would benefit from being simplified. I would recommend only showing the WT, each of the six deletions, and the epistatic substitution(s) that restored each mutation genotype back to WT charging level. The rest would be better suited as a supplemental figure.

Thank you for this suggestion. We rearranged the figure accordingly and moved some of the data to Supplementary Figure 4.

3) The paper concludes (lines 264-267) with a fairly specific order of events. The idea presented in the final paragraph of the manuscript is that mutational burden increased, and deleterious mutations in tRNAs selected for the evolution of aaRS recognition. However, it is also possible that the aaRS recognition

evolved first and permitted the degradation of tRNA sequence via nearly-neutral divergence. The authors should consider being more circumspect in their explanation.

Indeed, our intention was to leave the order of events open. Thus, giving the impression that degeneration of mt-tRNA necessarily preceded the evolution of an altered recognition mechanism was not our intention. Instead, we indeed think that the rapid evolution of mt tRNAs is a consequence of the changed recognition mode by mt aaRSs. We thank the reviewer for pointing this out. We changed passages in the discussion accordingly (p. 14-15).

4) It would be beneficial to include some aspects of mitochondrial inheritance that differ from what is assumed under most cases of Muller's ratchet. For example, many cells contain multiple mitochondria, and there are quality control mechanisms including purifying selection to reduce the chances of transmitting harmful mtDNA to offspring (e.g. reviewed in Mishra and Chan, Nature Reviews, 2014). Differences in mitochondrial quality control across species may impact your model, and should be discussed.

Thank you for this suggestion and for bringing this literature to our attention. We were of course aware of recent advances in the understanding of quality control mechanisms in animals and the use of drastic bottlenecks and strong purifying selection during germ cell development. Interestingly, however, it was found that in mammals these mechanisms are primarily acting to purge non-synonymous mutations in protein-coding regions, while they are curiously inefficient at removing mutations in mt tRNA genes, especially at low heteroplasmic levels (e.g. Stewart, et al. PLoS Biol 2008 6:e10). This suggests that mutations in mt tRNAs can more easily bypass the dedicated quality control mechanisms, which is consistent with the high frequency of mt tRNA polymorphisms in human populations (e.g. McFarland, et al., Trends Genet 2004, 20:591-6; Ruiz-Pesini & Wallace, Human Mutation 2006, 27(11): 1072-1081) and the high nucleotide substitution rates of mt tRNAs that we observe even between closely related animal species. It is interesting to note that in invertebrates (Drosophila) the selective sieve appears to be wider than in mammals due to a less stringent bottleneck and weaker purifying selection, which allows even deleterious mtDNA mutations to remain in the population significantly longer than observed e.g. in mice (Stewart, et al. PLoS Biol 2008 6:e10; Stewart & Larsson, PLoS Genet 2014, 10:e1004670). This could possibly be a contributing factor to the higher substitution rates and stronger degeneration of mt tRNAs in invertebrate lineages that results in the peak-like distribution for the divergence between protostomes and deuterostomes (Figure 1a). Moreover, the reduced sequence-dependency in mitochondrial aaRS/tRNA systems likely also contribute to the relaxed selective regime against mt tRNA sequences, which, as we suggest, plays an important role to allow their high substitution rates in bilaterian mitochondria.

In summary, we agree that the peculiarities of mitochondrial inheritance and dedicated quality control mechanisms play an important role. Yet the mechanisms in place to prevent deleterious mtDNA mutations to be transmitted between generations appear to be less active on mt tRNA genes and in any case does not prevent the accumulation of mutations, structural degeneration and loss of identity determinants. As this mutation accumulation and degeneration occurs in spite of quality control mechanisms, bottlenecks and purifying selection, we felt that an extended discussion may lead too far away from the main argument, which is primarily mechanistic in nature, and focused on the question how specificity is achieved in aminoacylation systems in spite of the inefficiency of purifying selection in mt tRNAs (our model may be viewed as a complementary molecular mechanism to maintain mitochondrial quality to cope with those mutations that escape germ cell selection). We hope that the reviewer understands our decision to keep the discussion as concise as possible and thus avoid an excursion into mitochondrial inheritance. If the reviewer should however prefer that these points are addressed, we will be happy to include it either in the main text or as supplementary discussion.

We thank the three reviewers for providing valuable comments and suggestions on our manuscript, which improved the clarity and depth of the presentations.

REVIEWERS' COMMENTS:

Reviewer #1 (Remarks to the Author):

The authors have now addressed all my concerns and toned down the claims substantially, as suggested!

Reviewer #3 (Remarks to the Author):

The authors have thoroughly addressed all of my concerns and I recommend publication.
